# TMBIM6 enhances dopaminergic neuron survival by modulating the IRE1a pathway in Parkinson’s disease

**DOI:** 10.1038/s41419-025-08391-5

**Published:** 2026-04-03

**Authors:** Pablo Ahumada-Montalva, Francisco Muñoz-Carvajal, Sara Bórquez-Macaya, Nohela Arévalo-Ramírez, Marisol Cisternas-Olmedo, Carolina Jerez, Valentina Urbina-Muñoz, Wileidy Gomez, Pamela J. Urrutia, Etienne C. Hirsch, Manuel Ahumada, Pablo Barrias, Camila Muñoz-Yañez, Ariel Herrera-Vásquez, Gloria Arriagada, Ute Woehlbier, Melissa Calegaro-Nassif, Mario Sanhueza, Rene L. Vidal, Diego Rojas-Rivera

**Affiliations:** 1 Cell Death & Biomedicine Lab, Center for Biomedicine, Universidad Mayor, Santiago, Chile; 2 Translational Neurobiology Lab, Center for Integrative Biology, Universidad Mayor, Santiago, Chile; 3 Center for Geroscience, Brain Health and Metabolism, Santiago, Chile; 4 Center for Integrative Biology, Universidad Mayor, Santiago, Chile; 5 Department of Neurology, The University of Texas Health Science Center at Houston, Houston, USA; 6 Autophagy and Neuroprotection Lab, Center for Biomedicine, Universidad Mayor, Santiago, Chile; 7 Resilient Aging Lab, Institute for Nutrition & Food Technology (INTA), Universidad de Chile, Santiago, Chile; 8 Sorbonne Université, Institut du Cerveau—ICM, Paris, France; 9 Center for Applied Nanotechnology, Universidad Mayor, Santiago, Chile; 10 Centro de Biotecnología Vegetal, Facultad de Ciencias de la Vida, Universidad Andrés Bello, Santiago, Chile; 11 Instituto de Ciencias Biomédicas, Facultad de Medicina, Universidad Andrés Bello, Santiago, Chile; 12 Center for Resilience, Adaptation and Mitigation, Universidad Mayor, Temuco, Chile

**Keywords:** Apoptosis, Parkinson's disease, Mechanisms of disease, Cell death in the nervous system, Neurodegeneration

## Abstract

The core pathological hallmark of Parkinson’s disease (PD) is the progressive degeneration of dopaminergic (DAergic) neurons in the substantia nigra pars compacta (SNpc), driven by misfolding and aggregation of a-synuclein (aSyn) into Lewy bodies. This triggers severe cellular dysfunction, including endoplasmic reticulum (ER) stress and the dysregulation of the unfolded protein response (UPR). TMBIM6, an anti-apoptotic ER protein, inhibits the UPR sensor IRE1a. Although TMBIM6 exhibits neuroprotective effects in neurological disorders, its role in PD-related DAergic neuron survival remains unknown. We report that TMBIM6 mRNA is increased in cellular models exposed to 6-hydroxydopamine (6-OHDA), rotenone, or aSyn preformed fibrils (PFFs), whereas TMBIM6 protein levels are elevated in postmortem PD SNpc, indicating translational relevance. Modulating TMBIM6 expression in DAergic cells and primary neurons showed that knockdown increased aSyn toxicity, while overexpression is protective. Single-cell RNA-seq analysis of PD SN revealed selective disruption of TMBIM6 co-expression with key UPR effectors (HSPA5, ERN1, and XBP1), and reduced TMBIM6 levels in vulnerable DAergic neurons. Mechanistically, TMBIM6 directly binds IRE1a, and aSyn PFFs disrupt this complex, leading to IRE1a activation; genetic or pharmacological IRE1a inhibition prevented cell death in TMBIM6-deficient cells. In vivo, TMBIM6 downregulation in *Drosophila melanogaster* worsens rotenone-induced DAergic neuron degeneration and motor impairments, while adeno-associated virus (AAV)-mediated TMBIM6 overexpression in mice improves motor function and neuron survival. Our results demonstrate that TMBIM6 modulates ER stress responses, promoting DAergic neuron survival by regulating IRE1a activity. Consequently, the TMBIM6/IRE1a axis represents a promising therapeutic target for mitigating neurodegeneration in PD and related disorders.

## Background

Parkinson’s disease (PD) is classified as a neurodegenerative disorder and is recognized as the second most common multifactorial neurodegenerative condition in the aging population [1]. Currently, there is no known cure for PD, which presents a significant public health challenge [2, 3]. The etiology of PD remains largely unknown, with most cases being sporadic and primarily linked to environmental factors [2, 3]. The primary clinical feature of PD is progressive impairment of voluntary motor control, including rigidity, bradykinesia, abnormal posture, impairment in motor control, and resting tremor, which are mainly caused by the gradual functional decline and cell death of dopaminergic (DAergic) neurons in the Substantia Nigra pars compacta (SNpc) of the midbrain [4, 5]. The evidence suggests that movement impairment results from decreased synaptic DAergic transmission in the motor region of the striatum, by the loss of their axon terminals in the striatal projection, and the DAergic neuron death [6–8]. The pathophysiology of PD involves various forms of cellular stress, which collectively lead to the apoptotic death of DAergic neurons [9, 10]. However, the precise mechanisms underlying these processes remain unclear. A key early event in PD is the toxic accumulation of misfolded alpha-Synuclein (aSyn) into Lewy Bodies (LBs), cytoplasmic protein aggregates that contribute to the progressive loss of SNpc DAergic neurons and the onset of motor deficits [11].

Inositol-Requiring Enzyme 1 Alpha (IRE1a), a key endoplasmic reticulum (ER) stress sensor and the main regulator of the unfolded protein response (UPR), controls the activation of XBP1 through its endoribonuclease activity, linking ER stress to adaptive or pro-apoptotic responses [12]. In this context, IRE1a has been suggested to play a pivotal role in driving the degeneration of DAergic neurons in PD models. Toxic forms of aSyn have also been shown to activate IRE1a, further driving neuronal dysfunction [12, 13]. Also, paraquat, a known neurotoxin, activates the IRE1a/ASK1/JNK signaling cascade, which is closely linked to apoptosis in various in vitro PD models [14]. Overexpression of ASK1 accelerates paraquat toxicity and apoptosis in cellular PD models [15], suggesting that IRE1a/ASK1/JNK drives cell death in PD. In vivo studies further corroborate this mechanism, showing that IRE1a is hyperactivated in response to aSyn accumulation in fly photoreceptor neurons. This hyperactivation leads to neuronal death through a mechanism independent of XBP1 but dependent on JNK signaling [13].

The transmembrane Bax inhibitor motif 6 (TMBIM6) was discovered in a functional screening in yeast as a human gene product that suppresses the BAX-induced apoptosis [16, 17]. The cellular localization of the TMBIM6 protein is in the ER membrane and interacts with various regulatory factors involved in cell death and ER stress [18]. The C-terminal domain of TMBIM6 binds to the ribosomal protein L19 (RPL19) and interacts with the cytosolic domain of IRE1a, thereby directly inhibiting IRE1a signaling and the subsequent splicing of XBP1 mRNA, regulating cell death [17, 19, 20]. TMBIM6 in the central nervous System (CNS) can be a protective factor in neurological disorders. Overexpression of TMBIM6 can inhibit the apoptosis of hippocampal neurons in rats with subarachnoid hemorrhage by inhibiting the activation of the IRE1a/JNK signaling pathway [21]. TMBIM6 also reduces early brain injury by attenuating apoptosis mediated by ER stress [22, 23]. These findings suggest that TMBIM6 has a neuroprotective role. However, its role and therapeutic potential in PD have not been clarified.

Here, we investigated the role of TMBIM6 in promoting DAergic neuron survival in PD models. We analyzed TMBIM6 expression in postmortem PD samples and found an overall increase in TMBIM6 levels in the SNpc. Re-analysis of public single-cell RNA-seq datasets from the SN of PD patients, however, revealed disrupted co-expression between TMBIM6 and IRE1a target genes; notably, TMBIM6 was selectively reduced in DAergic neurons identified as vulnerable to degeneration. We modulated TMBIM6 expression in both SN4741 cells and primary neurons exposed to neurotoxins that induce parkinsonism, such as 6-hydroxydopamine (6-OHDA), rotenone, and aSyn preformed fibrils (PFFs), and assessed cell death using a panel of cytotoxicity assays. Using two complementary approaches (siRNA-mediated knockdown of TMBIM6 and IRE1a expression, alongside pharmacologic inhibitors), we identified a contribution of IRE1/JNK pathways to TMBIM6-regulated cell death induced by aSyn. At the molecular level, we found that toxic aSyn species disrupt the TMBIM6/IRE1a complex, promoting its activation and downstream cell death signaling. Finally, to assess the in vivo relevance of TMBIM6 for PD, we employed two complementary strategies. First, taking advantage of the high evolutionary conservation of the TMBIM family in *Drosophila melanogaster* (*D. melanogaster*) and its widespread use as a PD model, TMBIM6 knockdown led to DAergic neuron degeneration and exacerbated the rotenone-induced motor phenotype, a widely used PD model. Second, through adeno-associated virus (AAV), we expressed human TMBIM6 in the SNpc of mice exposed to 6-OHDA. TMBIM6 overexpression protected DAergic neurons from 6-OHDA-induced cell death and prevented motor deficits.

Taken together, findings suggest that TMBIM6 exerts a neuroprotective effect in PD, regulating the IRE1a/JNK signaling pathway to promote DAergic neuron survival. These findings highlight TMBIM6 as a potential therapeutic target to prevent DAergic neuron loss and motor impairment, as well as to slow the progression of PD.

## Materials and methods

### In silico TMBIM6 expression analysis

The levels of *TMBIM6* mRNA expression in human CNS were determined using available data from The Human Protein Atlas database. We analyzed tissue data for normalized RNA expression generated by the FANTOM5 project [24]. The levels of *TMBIM6* mRNA expression in postmortem SN were determined using data from postmortem neurodegenerative studies available in the NCBI/GEO database. This dataset is accessible under the number GSE8397 [25] and encompasses postmortem medial SN transcriptomic profiles. A list of known TMBIM6 interactors was generated and analyzed using Ingenuity Pathway Analysis (IPA; QIAGEN Inc., https://digitalinsights.qiagen.com/IPA) [26]. Core analysis was performed with the following settings: (i) direct relationships between molecules, (ii) based on experimentally observed data, and (iii) all data sources were admitted from the Ingenuity Knowledge Base.

### Human brain tissue dissection

All experiments on human autopsy brain tissue were carried out following approval from the Institutional Human Ethics Committee, and all guidelines were followed (Institutional Human Ethics Committee Approval 4/2010). For the dissection of the SNpc, cryostat-cut sections of flash-frozen human midbrain tissue from PD patients and age-matched controls were obtained from ICM Brain Bank and the Neurocen Pitié-Salpêtrière hospital, Paris. Control individuals had no history of neurological or psychiatric disorders. PD patients exhibited characteristic motor symptoms, including akinesia, rigidity, and/or resting tremor. All PD cases had been treated with DAergic agonists and/or L-DOPA and had current or prior evidence of dyskinesia. The clinical diagnosis was confirmed by neuropathological examination, which revealed the presence of Lewy bodies in the SN. For Western blot analysis, samples from 10 PD patients and 9 age-matched controls were examined. The average age at death was 78.1 ± 6.2 years for PD patients and 82 ± 5.2 years for controls, with comparable postmortem delays (26 ± 13.7 h and 17.3 ± 8.9 h, respectively). Clinical and demographic data are shown in Fig. S3A, B. The SNpc tissue was carefully handled on dry ice throughout the procedure. Following dissection, the tissue was cut into smaller fragments and homogenized in RIPA buffer supplemented with protease inhibitors, using a 1:10 ratio of tissue weight to buffer volume. Protein extraction was performed under cold conditions to maintain protein integrity for subsequent Western blot analysis.

### PD cohort and peripheral blood mononuclear cells (PBMC) sampling

These experiments were performed at the Neurology Department of The Hospital Clínico Fuerza Aérea de Chile (FACH) and at the CIB of Universidad Mayor in Santiago, Chile, in accordance with the Declaration of Helsinki. They were approved by the local ethics committee of the FACH Hospital. All study participants, including 16 PD patients and 13 healthy controls, provided signed written informed consent prior to their enrollment. PD patients were evaluated through their clinical history, a physical examination, and scoring using the modified Hoehn and Yahr scale (H&Y) [27], and the Unified PD Rating Scale (UPDRS) [28]. Eligibility criteria included patients aged 18 years or older, with a documented history of follow-up at the FACH hospital, and being in stages 1–4 of PD according to the modified H&Y scale. Exclusion criteria included patients younger than 18 years or diagnosed with stage 5 PD. The venous blood samples analyzed were obtained from 13 healthy individuals (6 male, 7 female) and 16 PD patients (8 male, 8 female). Detailed demographic and clinical characteristics—including age, sex, H&Y stage, UPDRS-III score, and years since diagnosis—are provided in Supplementary Fig. S4A, B. The venous blood samples (30 ml per participant) were collected at the FACH Hospital using ethylenediaminetetraacetic acid (EDTA) tubes. Plasma and PBMC fractions were isolated using a centrifugation-based protocol with a ficoll-hypaque gradient, following established methods [29].

### Cell culture and primary cortical neuron (PCN) culture

N2a and SN4741 cells were cultured and maintained in DMEM high-glucose medium supplemented with 5% fetal bovine serum (Life Technologies, cat. 11965092) at 37 °C in a humidified incubator with 5% CO_2_. PCNs were obtained from C57BL6 wild-type mice at 17 days of embryonic development. Briefly, the embryonic brain cortices were isolated and homogenized in a high-glucose DMEM medium. The tissue was then incubated with 0.25% trypsin (Corning, Mediatech, Inc., Manassas, VA, USA) for 10 min at 37 °C and mechanically disaggregated to obtain isolated PCNs. Then, the PCNs were centrifuged at 10,000 × *g* for 10 min at room temperature. Isolated PCNs were cultured in a high-glucose DMEM medium supplemented with 10% FBS. After 24 h, the PCNs were maintained in Neurobasal Medium (Gibco, Thermo Fisher Scientific, Waltham, MS, USA) supplemented with B-27 complex (Gibco, Thermo Fisher Scientific, Waltham, MS, USA) at 37 °C in a humidified incubator with 5% CO_2_ until the end of the experiments.

### RNA extraction and Reverse transcription-quantitative real-time (qRT-)PCR

Total RNA was extracted from N2a cells, SN4741 cells, PCN cultures, human blood samples, and *D. melanogaster* head, using TRIzol reagent following the manufacturer’s instructions (Invitrogen, Carlsbad, CA, USA), and 2 μg of RNA was used to generate cDNA with the High-Capacity cDNA reverse transcription kit (Applied Biosystem, Thermo Fisher Scientific, Waltham, MA, USA) according to the manufacturer’s protocol. qRT-PCR was performed using the SYBR Green Reagent Kit (Applied Biosystems, Foster City, CA, USA) on an ABI PRISM 7700 Sequence Detection System under the following conditions: 95 °C for 5 min, followed by 40 cycles of 94 °C for 10 s, 51–55 °C for 10 s, and 72 °C for 30 s. Reactions were performed in duplicate runs for each sample, and mouse genes were normalized to the level of b-actin or RPL-19 housekeeping genes; human genes were normalized to the level of 18S and hSHDA housekeeping genes; *D. melanogaster* genes were normalized to the level of 18S housekeeping gene. The primers were purchased from OligoTM (Macrogen, Geumcheon-gu, Seoul) (Fig. S1-A–C).

### PD in vitro model

To mimic sporadic PD in cellular models, cells were exposed to parkinsonism stimulus (6-OHDA or rotenone). The stock solution of 6-OHDA (Sigma-Aldrich, St. Louis, MO, USA) was previously dissolved in 0.02% ascorbic acid, and 50 μM 6-OHDA was incubated directly in cell cultures for 24 h. Rotenone (Sigma-Aldrich, Saint Louis, MO, USA) was dissolved in DMSO, and 25 μM was incubated directly in the cell cultures for 24 h. Also, cells were exposed to recombinant aSyn PFFs [30]. Briefly, the mouse aSyn monomers (Mons) were dissolved in PBS, with the pH adjusted to 7.5 and constant agitation (1000 rpm) for 7 days at 37 °C. Next, the pellet containing the insoluble PFFs was separated by sonication (20% amplitude, 5-s pulse on, and 5-s pulse off, repeated four times on ice) to obtain smaller seeds, aliquoted and stored at −80 °C until use. SN4741 cells were exposed to 10 μM aSyn Mons or 10 μM aSyn PFFs for 24 h. PCNs were exposed to 10 μM aSyn Mons or PFFs for 7 days. PBS was used as a control. To quantify aSyn aggregates in SN4741 cells, total protein extracts were prepared and analyzed by SDS–PAGE and Western blot. High-molecular-weight (HMW) aSyn species were defined as immunoreactive signal migrating above ~35 kDa up to the stacking region. Membranes were imaged within the linear exposure range and the HMW signal was quantified in ImageJ by measuring the integrated density of the HMW region after subtracting the local background. For knockdown experiments, values were normalized to β-Tubulin. For overexpression experiments, total protein per lane was assessed by stain-free imaging using 2,2,2-trichloroethanol (TCE)-activated gels, and densitometric values were normalized to this TCE total-protein signal for each lane.

### Mitochondrial membrane potential measurement (ΔΨm)

ΔΨm was assessed by incubating cells with 200 nM of 3,3’-Dihexyloxacarbocyanine Iodide (DIOC(6)3) for 30 min at 37 °C. Next, the DIOC(6)3 solution was removed, and the fluorescence signal of DIOC (6)3 retention was measured using a FLUOstar multi-mode microplate reader (FLUOstar Omega, BMG LabTech). 1 μM of Carbonyl cyanide 4-(trifluoromethoxy)phenylhydrazone (FCCP) (Merck KGaA, Darmstadt, Germany) was used as a positive control for the loss of ΔΨm.

### Cell death assays

The activity of caspase-3 was determined by the fluorescence intensity of DEVD-AMC [31]. Cells were incubated with 15 μM DEVD-AMC for the same duration as the cell death stimulus, and the fluorescence signal of DEVD-AMC was measured using a FLUOstar multi-mode microplate reader. 2 μM Staurosporine was used as a positive internal control of caspase 3 activity (data not shown). SN4741 cell viability was measured by the MTT Cell Proliferation Assay Kit (BioVision, Cat. K299, CA, USA) according to the manufacturer’s instructions after 24 h of incubation with the cell death stimulus [30]. Cytotoxicity was measured by LDH release using the CyQUANT^TM^ LDH Cytotoxicity assay kit (Invitrogen, Cat. C20301, Waltham, MA, USA) following the manufacturer’s instructions. Absorbance from LDH release was measured using a FLUOstar multi-mode microplate reader after 24 or 48 h of incubation with cell death stimulus [30].

### Downregulation of TMBIM6 in vitro

For transient downregulation of TMBIM6, 2 × 10^5^ SN4741 cells were seeded in 32 mm^2^ dishes and cultured until they reached 70% confluence. Then, siRNA targeting mouse TMBIM6 (Dharmacon^TM^, Horizon Discovery, Waterbeach, UK) was transfected using Lipofectamine^TM^ RNAiMAX (Invitrogen, Carlsbad, CA, USA) according to the manufacturer’s instructions. Effective downregulation was assessed by RT-qPCR or Western blot 48 h after transfection. For this, proteins were extracted, and electrophoresis was performed using 12% bis-acrylamide gel. Proteins were transferred to a PVDF membrane, and immunodetection was performed using a mouse anti-TMBIM6 antibody (Santa Cruz Biotechnology, Cat. Sc-73483, TH, USA) overnight at 4 °C. The PVDF membrane was then incubated with mouse-HRP conjugated antibody (Jackson ImmunoResearch Laboratories, Cat. AB2313567, Inc., PA, USA). Membranes were developed using a ChemiDoc photodocumenter (Bio-Rad Laboratories, Inc., Hercules, CA, USA).

### *D. melanogaster* strains and culture

The Fly stocks used in this study (Fig S1-D) include Elav-Gal4 (BL#8765), GMR-Gal4 (BL#1104), Ple-gal4 (BL#8848), and siControl (si*Cntrl*, UAS-TRIP, BL#35787), which were obtained from the Bloomington Drosophila Stock Center (BDSC); as well as UAS-RNAi-dTmbim6 (VDRC#37108) [19, 32–35] from the Vienna Drosophila Resource Center (VDRC). All fly stocks were maintained on standard *D. melanogaster* medium, consisting of 112.5 g of molasses, 35 g of dry yeast, 90 g of corn flour, 9 g of agar, 2.5 g of the antifungal agent Tegosept (diluted in 10 mL of 95% ethanol), and 6 mL of propionic acid per 1 L of water. Flies were kept at 25 °C under a 12-h light/12-h dark circadian cycle.

### *D. melanogaster* eye development scanning

For eye development assays, flies were maintained at 25 °C. Upon eclosion, twenty-five female flies per genotype were collected and aged for 5 days. They were then frozen at −20 °C for 2 h before imaging. A single image per fly was captured using image acquisition software and analyzed against a neurodegeneration scale, where a score of 5 represents optimal eye integrity and 0 indicates severe degeneration (Fig. S5A, B). Each photograph was evaluated blindly by three individuals, and the assigned scores were used to calculate the mean eye integrity score and frequency distribution across categories.

### Modeling PD *D. melanogaster*

In *D. melanogaster*, the PD model was generated using rotenone (Sigma-Aldrich, St. Louis, MO, USA, Cat. R8875). Rotenone was dissolved in DMSO and added to the fly medium at a final concentration of 300 μM. 10 dpe flies were exposed to rotenone for 7 days, with the solution refreshed every 48 h. DMSO was used as a control. After the treatment, motor performance was assessed, and fly brains were isolated and fixed for immunofluorescence analysis.

### Motor assay in *D. melanogaster*

For the climbing assay, groups of 10 female flies per genotype were placed at the bottom of clear vials by gentle tapping. The number of flies that climbed 10 cm within 10 s was recorded using the automated *D. melanogaster* activity monitoring system (DAM System, Trikinetics). Flies underwent three training sessions before the assay, and the experiment was repeated 10 times per time point. For the spontaneous activity assay, groups of 10 female flies per genotype were placed in horizontal vials, and their movement was tracked for 24 h. The number of times the flies interrupted the laser sensor was recorded using the DAM System (Trikinetics). Differences in peak activity were analyzed.

### Immunostaining for TH^+^ cells in *D. melanogaster*

Tyrosine hydroxylase-positive (TH+) cells in *D. melanogaster* brains were labeled using a rabbit anti-TH antibody (1:400; Merck Millipore, cat. AB152) overnight at 4 °C and then incubated with goat anti-rabbit 488 secondary antibody (1:400). The number of TH+ cells in the DAergic clusters [36] from the brains of female flies were quantified using confocal microscopy, along with cell diameter measurements.

### Expression of human TMBIM6 in vitro

Lentiviral particles that allow stable expression of human TMBIM6 and TMBIM6^D213A^ with an HA tag (TMBIM6^HA^ and TMBIM6^D213A/HA^, respectively) were generated using a third-generation lentiviral system. The lentiviral constructs were designed and purchased from Vector Builder Company (Chicago, IL, USA). Briefly, lentiviral particles were produced by co-transfecting HEK293T cells with the transfer vector, packing plasmids, and an envelope vector (pseudo-typed with VSV-G) using Lipofectamine™ 2000 (Invitrogen, Carlsbad, CA, USA). The supernatant containing the lentiviral particles was collected 48 h post-transfection and filtered through a 0.45-µm membrane. Then, 5 × 104 SN4741 cells were transduced with 4 mL supernatant in the presence of Polybrene, and 48 h later, the cells were selected with 10 µM puromycin. For transient expression, 2.5 × 10^5^ SN4741 cells were seeded in 32 mm^2^ dishes, and 24 h later, the cells were transfected using TransIT-X2^TM^ (Mirus, Cat. 6006) according to the manufacturer’s instructions. An empty vector (Mock) was used as a control for both stable and transient TMBIM6^HA^ expression. Effective expression was assessed by Western blot after 48 h of transfection.

### Single-nucleus RNA sequencing data and correlation analysis

We analyzed publicly available single-nucleus RNA sequencing (snRNA-seq) data from human post-mortem SN tissue, obtained from both healthy individuals and patients with PD. The dataset (GEO accession: GSE178265) was originally generated and published by Kamath et al. [37], and includes high-resolution transcriptomic profiling of 21 human donors (10 healthy controls and 11 PD cases), resulting in 18,399 high-quality nuclei after quality control filtering. The dataset was downloaded in *gct* format from the Broad Institute’s Single Cell Portal (SCP1768). Expression values for each gene were log-normalized and processed as provided by the authors. We focused our analysis on five genes: *TMBIM6* and four UPR-related genes (*HSPA5, ERN1, XBP1*, and *BLOC1S1*), selected based on their known roles in ER stress and their documented involvement in UPR signaling pathways.

To investigate cell-type-specific expression changes, we first subsetted DAergic neuron nuclei into “vulnerable” and “resistant” populations, as defined by the original authors using *SOX6*^*+*^*/AGTR1*^*+*^ and *CALB1*^*+*^*/GEM*^*+*^*, CALB1*^*+*^*/TRHR*^*+*^*, CALB1*^*+*^*/RBP4*^*+*^ as markers gene signatures, respectively³⁹. We then performed differential expression analysis between these two populations within the PD patient cohort. Statistical significance was determined using the Model-based Analysis of Single-cell Transcriptomics (MAST) test, a regression-based framework tailored for sparse single-cell expression data. Gene expression patterns were visualized using dot plots, where dot size represents the percentage of cells expressing the gene and color intensity corresponds to the average expression level in expressing cells.

To examine changes in co-expression patterns, we computed pairwise Pearson correlation coefficients between TMBIM6 and each of the four UPR genes using the log-normalized expression matrix. Correlations were calculated separately for nuclei derived from healthy and PD samples. Sample metadata, including disease condition, donor ID, and sex, was extracted from the accompanying annotation files and matched to the expression matrix by sample ID. To statistically evaluate whether the strength of correlation between TMBIM6 and each UPR gene differed significantly between conditions, we applied Fisher’s Z-transformation to the correlation coefficients. This method converts Pearson correlation values into normally distributed Z-scores, allowing direct comparison between two independent correlation estimates. The delta correlation (Δr) for each gene pair was calculated as the difference between the correlation coefficients in PD and healthy groups (Δr = r_PD_ − r_Healthy_). Raw p-values from the Z-test were subsequently adjusted for multiple hypothesis testing using the Benjamini-Hochberg False Discovery Rate (FDR) correction method. An FDR-adjusted p-value threshold of < 0.05 was used to determine statistical significance. All statistical analyses were performed in R (v4.3.1) using the stats, dplyr, scales, MAST and ggplot2 packages. Plots and data processing scripts are available upon request.

### Proximity ligand assay (PLA)

SN4741-Mock, SN4741-TMBIM6^HA^ and SN4741-TMBIM6^D213A/HA^ cells were fixed with 4% paraformaldehyde and blocked with the blocking solution supplied by the Duolink^TM^ PLA kit (Merck KGaA, Darmstadt, Germany) according to the manufacturer’s instructions. Briefly, the fixed cells were incubated overnight with an anti-HA antibody (Invitrogen-Thermo, Cat. 21683, Waltham, MA, USA) and anti-IRE1a antibody (Cell Signaling Technology, Cat. 3294S, Danvers, MA, USA). After 3 washings, cells were incubated successively with PLA probes, ligation solution, and amplification solution at 37 °C. Coverslips were mounted, and the images were examined using a confocal microscope [31].

### Inhibitor reagents

The RNase activity of IRE1a was inhibited with 10 µM 4µ8C or 10 µM MKC (MedChemExpress, Monmouth Junction, NJ, USA). PERK activity was inhibited with 10 nM GSK2606414 Perk inhibitor (PERK-inh) (Merck KGaA, Darmstadt, Germany). JNK activity was inhibited with 10 µM AS601245 (JNK-inh)(MedChemExpress, Monmouth Junction, NJ, USA). BAX activity was inhibited with 10 µm Bax inhibitor (Bax-inh) (MedChemExpress, Monmouth Junction, NJ, USA), and caspase activity was inhibited with 20 µm ZVAD_-_FMK (Casp-inh)(MedChemExpress, Monmouth Junction, NJ, USA).

### Solid-phase peptide synthesis

TMBIM6^C-term^ mimic peptide (RRRRRRRRLAMNEKDKKKEKK) and its scramble sequence (RRRRRRRRKEKMKLKANKEDK) were synthesized using solid-phase peptide synthesis (SPPS) with the Fmoc protecting group strategy, based on 2-chlorotrityl resin (Fig. S9). The resin was swollen with dry dichloromethane (DCM) for 30 min under constant shaking (x2). The coupling of the first amino acid was done by mixing the swollen resin with 1.5 equivalents of Fmoc-Lys(Boc)-OH in 2 mL of dry DCM and Diisopropylethylamine (DIPEA, 3 equivalents) at RT for 90 min. Then, the resin was washed with dry DCM three times. The unreactive sites were capped with a mixture of DCM/MeOH/DIPEA (85:10:5) at room temperature for 30 min. The resin was washed with DCM twice and with Dimethylformamide (DMF) four times. Later, Fmoc deprotection was performed using a 20% Piperidine solution in DMF at room temperature for 10 min. Then, the peptide chain was elongated by coupling the corresponding amino acid (5 equivalents) in the presence of hexafluorophosphate benzotriazole tetramethyl uronium (HBTU; 5 equivalents) as the activator and DIPEA (10 equivalents) at RT for 45 min for each amino acid. For each Fmoc-Arg(Pbf)-OH, the coupling step was performed twice using HATU (hexafluorophosphate azabenztriazole tetramethyl uronium) as the activator. All deprotection and coupling steps were monitored by the Chloranil Test. After completing the desired peptide sequence, the peptide was cleaved from the resin with a mixture of trifluoroacetic acid (TFA)/thioanisole/(ethylenedioxy) diethanethiol (DODT)/anisole (88/5/5/2) at room temperature for 4 h. The peptide was then precipitated in cold diethyl ether and washed twice with the same solvent. The precipitated peptide was resuspended in a 0.1% TFA water solution and freeze-dried (Telstar Lyoquest at −85 °C). Crude peptides were purified using preparative HPLC coupled to a DAD detector (preparative HPLC-DAD, Jasco LC-4000 series) with a C18 column (150 × 4.6 mm, 130 Å, 3 µm, InertSil ODS-4 GL Sciences) as the stationary phase. The purification employed a gradient elution of solution A (0.1% TFA in H_2_O) and solution B (acetonitrile/H_2_O (60/40) with 0.1% TFA). Then, the purified peptide was freeze-dried overnight, and the precipitate was resuspended in a 100 mM HCl solution and freeze-dried again overnight. Peptides were analyzed and identified using UHPLC-MS/MS (Ultimate 3000 RSLC system coupled with a Linear Ion Trap Mass Spectrometer LTQ XL, Thermo Scientific) with an Inertsil® ODS-4 (3 μm, 2.1 × 100 mm, GL Sciences) column, employing a gradient elution of solution A (0.1% Formic Acid in H_2_O) and solution B (0,1% Formic Acid in acetonitrile/H_2_O (60/40) mixture).

### Adeno-associated viral vector in vitro

AAV vectors were designed and purchased from VectorBuilder Company. The control virus expresses only green fluorescent protein (GFP) (AAV-Mock^GFP^), meanwhile the experimental virus expresses TMBIM6^HA^ and GFP in a bicistronic manner mediated by an internal ribosomal entry site -IRES-sequence (AAV-TMBIM6^HA/GFP^). The PCNs were directly transduced with the AAV-TMBIM6^HA/GFP^ or AAV-Mock^GFP^ as a control, and transduction was assessed by western blot assay after 48 h. Afterward, the transduced cells were exposed to 50 µM 6-OHDA or 10 µM aSyn PFFs, and cell death was determined. 10 µM aSyn Mons and PBS were used as controls.

### Adeno-associated viral vector in vivo

Adult female mice (2-month-old) were injected with 2 µl of AAV-TMBIM6^HA/GFP^ or AAV-Mock^GFP^ into bilateral SN by stereotaxic surgery using coordinates from the atlas of Franklin and Paxinos, Second Edition, 2001: AP: −0.29 mm, LAT: -0.13 mm, DV: -0.42 mm. After 2 weeks post-injection, mice were injected with 5 µg/µL or 8 µg/µL of 6-OHDA in the unilateral striatum area (CPu) to generate an in vivo PD model, following coordinates from the atlas of Franklin and Paxinos, Second Edition, 2001: AP: +0.07 mm, LAT: -0.17 mm, DV: -0.31 mm. The injection volume was 2 µl per site; therefore, the total doses delivered were 10 µg (5 µg/µl × 2 µl) or 16 µg (8 µg/µl × 2 µl).

### Motor performance in mice

Motor performance in mice was evaluated by two different tests performed at 2, 3, 4, and 5 weeks post-injection (wpi) of AAV vectors. In the cylinder test, which measures forelimb use during explorative activity, mice were placed individually in a glass cylinder (11 cm in diameter, 20 cm in height). They were video recorded for 5 min using a camera. Only weight-bearing wall contacts made by each forelimb on the cylinder wall were scored. Wall exploration was expressed in terms of the % of impaired forelimb wall contacts relative to the total number of times the mouse touched the wall with one of the forelimbs [38]. Balance and coordination in mice were evaluated using the Beam test, which consists of recording the movement of the mouse on a narrow beam and determining the number of errors when placing a paw into a hole or losing balance [39].

### Statistical analysis

Differences between two groups were assessed using either an unpaired t-test or the Mann–Whitney U test. For comparisons involving more than two groups, the Kruskal–Wallis test followed by Dunn’s post-hoc test was used. Two-way ANOVA followed by Tukey’s multiple comparison test was performed when analyzing the effects of two independent variables. Unless otherwise stated, all experiments were performed with a minimum of three independent biological replicates (n = 3) per experimental group. For single-nucleus RNA sequencing (snRNA-seq) data, differential gene expression was assessed using the Model-based Analysis of Single-cell Transcriptomics (MAST) test, while differences in gene co-expression were evaluated by comparing Pearson correlation coefficients using Fisher’s Z-transformation. Statistical analyses were conducted using GraphPad Prism 9 (GraphPad Software, San Diego, USA) and RStudio (R version 4.3.2). The significance level was set at α = 0.05 and the statistical power at 1 − β = 0.80 (β = 0.20). Statistical significance was defined as: *=p < 0.05; **=p < 0.01; ***=p < 0.001; ****=p < 0.0001. A comprehensive summary of the statistical analyses corresponding to each figure is presented in Supplementary Statistics Table.

### Ethics approval and consent to participate

This study was conducted in accordance with the Declaration of Helsinki and approved by the Ethics Committee of Hospital Clínico Fuerza Aérea de Chile (FACH). All participants provided written informed consent before their inclusion in the study.

## Results

### TMBIM6 is dysregulated in response to PD-like stimuli

**Fig. 1:**
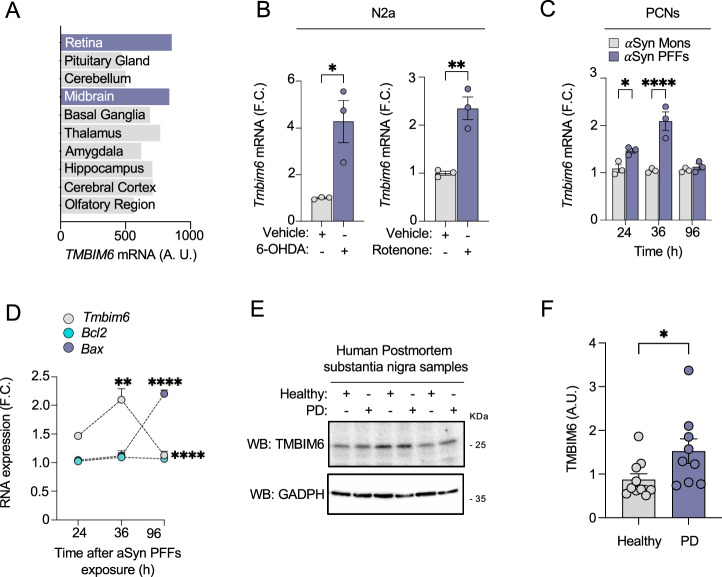
Regional expression profile of TMBIM6 in the human brain and its regulation in PD. **A** In silico *TMBIM6* mRNA expression in human CNS from THPA database. **B** *Tmbim6* mRNA expression on N2a cells after 18 h of exposure to 25 μM 6-OHDA or 50 μM rotenone. **C** *Tmbim6* mRNA levels over time in PCNs exposed to aSyn for 96 h. **D** Changes of expression of *Tmbim6*, *BcL2*, and *Bax* over time in PCNs exposed to aSyn for 96 h. **E** Representative Western blots of total protein extracts from postmortem human SN from neurologically healthy controls and PD patients, probed for TMBIM6 and GAPDH (loading control). Full, uncropped blots are provided in Supplementary Material. **F** Densitometric quantification of TMBIM6 from blots in (**E**). Band intensities were normalized to GAPDH for each lane; individual data points are shown with mean ± SEM (n = 9–10 per group). For experiments involving more than two groups, a two-way ANOVA followed by Tukey’s multiple comparison test was performed. Pairwise comparisons between two groups were analyzed using unpaired t-test (**B**) or the Mann–Whitney U test (**F**). All bars represent mean ± SEM. Statistical significance (p < 0.05) between samples is indicated in the figures as follows: *=p < 0.05; **=p < 0.01; ***=p < 0.001; ****=p < 0.0001.

We aimed to assess the neuroprotective role of TMBIM6 in PD, hypothesizing that TMBIM6 is involved in the survival of neurons, specifically in DAergic neurons, the primary cell type affected in PD pathology. To explore this, we first investigated whether TMBIM6 is expressed in DAergic neurons. Using data on *TMBIM6* mRNA expression levels from The Human Protein Atlas database (THPA) [24], we confirmed through in silico analysis that *TMBIM6* is expressed in various cortical regions of the human brain, with notably high expression in the midbrain and retina, two areas rich in DAergic cells [40, 41] (Fig. 1A). Considering that TMBIM6 is expressed in DAergic regions affected in PD pathology, we investigated whether TMBIM6 is involved in the cellular response to PD stimuli that cause DAergic neuron damage. We explored *Tmbim6* mRNA expression in response to a PD model using N2a cells exposed to a non-lethal 6-OHDA dose (25 μM) or rotenone (50 μM) for 21 h. Both 6-OHDA and rotenone caused a significant increase in *Tmbim6* mRNA expression in the N2a cell line (Fig. 1B). Next, we treated PCNs with aSyn Mons or aSyn PFFs for 96 h, and we observed an increase in *mTmbim6* mRNA expression during the first 36 h treated with PFFs, but it decreased by 96 h (Fig. 1C). In addition, we observed that aSyn PFFs did not induce changes in *Bcl2* mRNA expression but significantly increased *B*ax mRNA at 96 h when *Tmbim6* mRNA was decreased (Fig. 1D and S2A, B). In this analysis, we did not observe a significant difference between the aSyn Mons condition and control cells treated with only PBS. These data indicate that TMBIM6 is an early response element to stress induced by PD-like stimuli. However, with the persistence of the toxic stimulus*, Tmbim6* mRNA diminishes, allowing for an increase in the mRNA expression of the apoptosis executor BAX.

Next, we assessed TMBIM6 expression in postmortem samples from PD patients and compared them with age-matched controls. We observed a significant increase in TMBIM6 protein levels in the PD samples (Fig. 1E, F and Fig. S3D). However, we did not find a positive correlation between TMBIM6 levels and disease progression (Fig. S3C). Growing evidence indicates that PD pathology impacts the peripheral system [42]. Thus, we analyzed human *TMBIM6* mRNA expression levels by RT-qPCR in PBMCs from a live cohort of PD patients at stages 1-4 according to the modified H&Y scale [27]. In this analysis, *TMBIM6* mRNA levels in PBMCs from PD patients were comparable to those in healthy controls (Fig. S4C). Additionally, no significant differences were observed when comparing men and women, nor was there a clear association between *TMBIM6* levels and disease stage (Hoehn–Yahr and UPDRS 3 scale) (Fig. S4E, F); nevertheless, *TMBIM6* levels showed a positive correlation with overall PD progression (Fig. S4G). Together, these results logically raise the question regarding the role of TMBIM6 in the mechanisms of DAergic neuron survival/death in PD.

### Downregulation of TMBIM6 leads to increased PD-like cellular damage

**Fig. 2:**
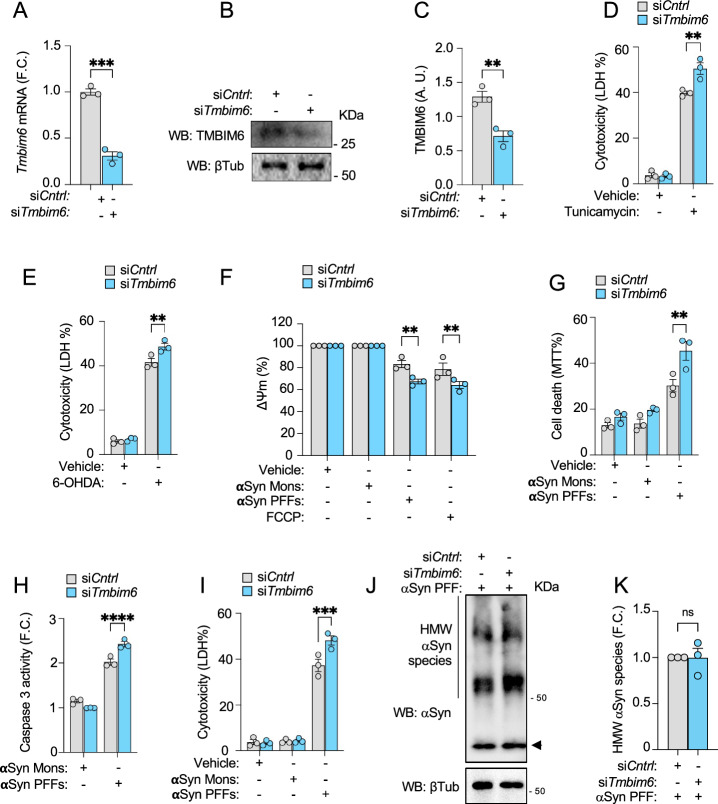
TMBIM6 knockdown sensitizes DAergic cells to aSyn PFF–induced degeneration. **A** Graph shows validation of decreased *Tmbim6* mRNA levels after 48 h of siRNA transfection in SN4741. **B**, **C** Representative immunoblots and quantification show *mTmbim6* KD cells after 48 h. **D** Cytotoxicity assay shows cell death induced by 10 μM Tunicamycin after 24 h in KD cells. Results are expressed as % of LDH release. **E** Cytotoxicity assay shows cell death induced by 50 μM 6-OHDA after 24 h in KD cells. **F** Retention of DiOC6(3) assay shows the effect of aSyn on ΔΨm in KD cells after 18 h. Results are expressed as % of DiOC6(3) retention. **G** MTT assay shows mitochondrial-dependent cell death induced by aSyn in KD cells after 24 h. Results are expressed as % of MTT. **H** DEVD-AMC fluorescent assay shows the effect of aSyn on Caspase-3 activity in *Tmbim6* KD cells after 24 h. Results are expressed as fold change of DEVD-AMC fluorescence intensity relative to vehicle. **I** Cytotoxicity assay shows the KD cell death induced by aSyn after 24 h. **J** Representative immunoblot of high–molecular-weight (HMW) aSyn species in SN4741 cells transfected with si*Cntrl* or si*Tmbim6* and treated with 10 µM aSyn PFFs for 24 h; Tubulin was used as a loading control. **K** Densitometric quantification of HMW aSyn bands from the experiment described in (**J**) (integrated density normalized to Tubulin). All bars represent mean ± SEM. For experiments involving more than two groups, a two-way ANOVA followed by Tukey’s multiple comparison test was performed. Pairwise comparisons between two groups were analyzed using unpaired t-test (**A**, **C**) or the Mann–Whitney U test (**K**). Statistical significance (p < 0.05) between samples is indicated in the figures as follows: *=p < 0.05; **=p < 0.01; ***=p < 0.001.

Building on the hypothesis that TMBIM6 is involved in the cellular response to PD-stimuli, we tested whether TMBIM6 is critical for supporting DAergic cell survival against cellular damage induced by PD-like neurotoxins. Thus, we generated a PD neurotoxic model in vitro in the presence or absence of TMBIM6. For this, we used the SN4741 cell line, an SN DAergic neuronal progenitor line that has been employed to clarify the mechanisms behind neurotoxicity in PD [30, 43]. We downregulated TMBIM6 expression by transfection of a siRNA targeting *Tmbim6* (si*Tmbim6*). A non-sense siRNA was used as a control (si*Cntrl*). The efficiency of si*Tmbim6* was determined by RT-qPCR and Western blot after 48 h, showing a significant reduction in TMBIM6 expression (Fig. 2A–C). To determine if TMBIM6 retains its anti-apoptotic activity in DAergic neurons and to understand the dynamic range of neuronal protection generated by TMBIM6 expression, we exposed si*Tmbim6*-transfected cells to the ER-stress inducer Tunicamycin. We observed a significant increase in cytotoxicity induced by 10 µM Tunicamycin in si*Tmbim6*-transfected DAergic cells compared to the control (Fig. 2D). Next, to explore whether TMBIM6 is involved in PD-like neurodegeneration, we performed a cytotoxicity assay in a pharmacologic in vitro PD model. 6-OHDA is widely used to mimic sporadic PD in in vitro and in vivo models, as it impacts mitochondrial complex I, inducing DAergic neuron death and recapitulating motor deficits [44]. We exposed si*Tmbim6*-transfected cells to 50 µM 6-OHDA and observed a significant increase in cytotoxicity in si*Tmbim6* cells compared to the control (Fig. 2E). These data indicate that TMBIM6 retains its anti-cell death activity in SN4741 cells against stimuli that independently impact the ER and mitochondria.

Next, we modeled PD in vitro by exposing cells to toxic aSyn species. Cells were treated for 24 h with 10 µM aSyn Mons (control), 10 µM aSyn PFFs (neurotoxic; most abundant protein in LBs [45]), or PBS (Vehicle), after which cell death was evaluated. Due to our previous observation that TMBIM6 expression impacts mitochondrial function, as does aSyn, we evaluated how TMBIM6 expression influences ΔΨm in response to toxic aSyn species. Using DIOC(6)3 dye to assess ΔΨm, we observed that aSyn PFFs, but no aSyn Mons, caused a loss of ΔΨm in si*Cntrl* cells, which drop is significantly pronounced in *Tmbim6*-deficient cells. FCCP was used as a control for ΔΨm loss (Fig. 2F). In addition, an MTT assay revealed that aSyn PFFs, but not Mons, significantly increase cell death in *Tmbim6*-deficient cells compared with controls (Fig. 2G). Because apoptosis has been identified as a possible cell death mechanism in PD, we used DEVD-AMC dye to determine caspase-3 activity. We observed that aSyn PFFs, but not aSyn Mons or vehicle, significantly increased caspase-3 activity in DAergic SN4741 cells lacking TMBIM6, in contrast with controls (Fig. 2H). These data indicate that TMBIM6 reduces the aSyn-induced apoptosis. Then, we analyzed the impact of *mTmbim6* deficiency on aSyn PFFs cytotoxicity. An LDH assay showed a significant increase in cytotoxicity induced by aSyn PFFs in *Tmbim6*-deficient cells compared with controls (Fig. 2I), without affecting the formation of HMW aSyn species (Fig. 2J, K). Together, these data suggest that TMBIM6 acts as an active apoptotic repressor and regulates PD-like neurotoxicity, without affecting aSyn aggregation. Furthermore, the downregulation of TMBIM6 increases cell damage induced by aSyn.

### Genetic downregulation of TMBIM6 in D. melanogaster causes DAergic neurodegeneration

**Fig. 3:**
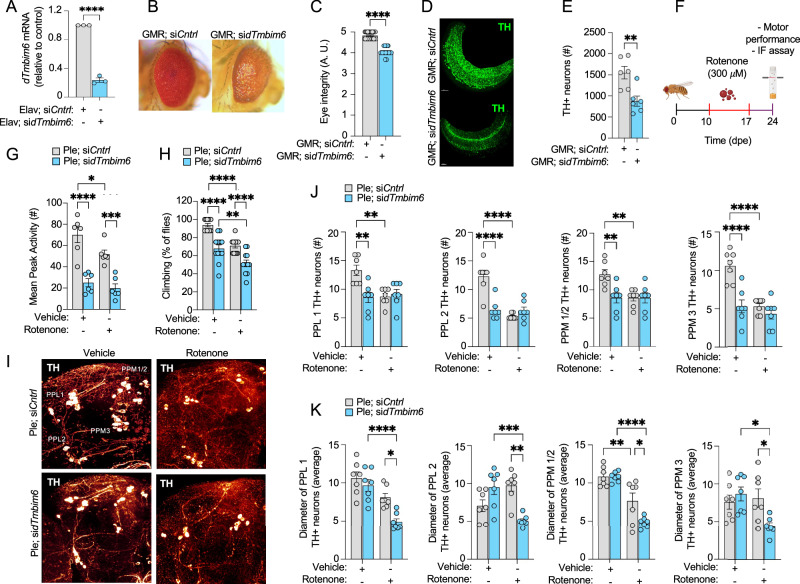
Effects of dTMBIM6 Knockdown in a *D. melanogaster* Pharmacological Model of PD. **A** d*Tmbim6* mRNA expression on homogenized flies’ heads. Results are expressed as fold change of mRNA expression. The bars represent mean ± SEM. **B** Optic image of eye integrity in RNAi-dTmbim6 flies incubated at 25 °C. **C** Quantification of eye integrity score in RNAi-*dTmbim6* flies incubated at 25 °C (n = 25 per group). **D** Immunostaining of TH+ neurons in the lamina of RNAi-dTmbim6 flies incubated at 25 °C (n = 6 per group). **E** Quantification of the number of TH+ neurons in the lamina of RNAi-dTmbim6 flies incubated at 25 °C (n = 6 per group). **F** Schematic representation of rotenone-induced PD model in *D. mel*. **G** Spontaneous activity in DAergic RNAi-dTmbim6 flies after exposition to rotenone 300 μM for 7 days (n = 6 populations of 12 flies). **H** Climbing assay showed the motor ability of DAergic RNAi-dTmbim6 flies exposed to rotenone 300 μM for 7 days (n = 12 per group). **I** Representative confocal images of IF assay showed TH+ cells from DAergic RNAi-dTmbim6 flies exposed to rotenone 300 μM for 7 days. **J** The numbers of TH+ cells were quantified in each DAergic cluster, and **K** the somal size was analyzed (n = 7 per group). All bars represent mean ± SEM. For experiments involving more than two groups, a two-way ANOVA followed by Tukey’s multiple comparison test was performed. Pairwise comparisons between two groups were analyzed using unpaired t-test (**A**, **C**) or the Mann–Whitney U test (**E**). Statistical significance (p < 0.05) between samples is indicated in the figures as follows: *=p < 0.05; ** = p < 0.01; ***= p < 0.001; ****= p < 0.0001.

Given that TMBIM6 is dysregulated in the acute response to PD, we investigated the contribution of TMBIM6 to cell survival and death mechanisms in neurons. We hypothesized that the loss of TMBIM6 leads to increased DAergic neuron death. To test this, we utilized the UAS-GAL4 system to drive TMBIM6 downregulation in *D. melanogaster* neurons. This system enables the tissue-specific expression of transgenes, allowing us to induce the expression of a validated UAS-siRNA targeting *D. melanogaster Tmbim6* (*dTmbim6*) in specific tissues [19, 32–35]. First, we validated the downregulation of *dTmbim6* mRNA in the entire nervous system of *D. melanogaster* using the Elav-GAL4 promoter. These analyses by RT-qPCR showed a significant decrease in *dTmbim6* mRNA levels (Fig. 3A). Next, to explore if the loss of TMBIM6 function impacts in vivo neuronal survival, we downregulated *dTmbim6* specifically in eye cells using the GMR-GAL4 promoter. In contrast to control flies (si*Cntrl*), the flies lacking *dTmbim6* in their eye cells exhibited a loss of pigmentation in the peripheral regions of the eye, as well as clusters of altered pigmentation in the center of the eye (Fig. 3B). We then assessed eye development by scoring eye integrity double-blindly, where 0 represents a total loss of eye integrity, and 5 corresponds to optimal eye morphology (Fig. S5A). These analyses revealed that, under 25 °C incubation conditions, flies lacking *dTmbim6* averaged a score of 4 on the eye integrity scale compared to controls (Fig. 3C). Interestingly, 80% of flies lacking *dTmbim6* at 25 °C scored 4 (Fig. S5B).

Since the lamina of *D. melanogaster* eye is rich in DAergic neurons [46], we performed immunostaining for TH+ neurons in the lamina of *D. melanogaster* eyes lacking *dTmbim6* or in *Cntrl* flies (Fig. 3D). We observed that in the absence of *dTmbim6*, the lamina of *D. melanogaster* shows a significant reduction in the number of TH+ neurons compared with controls (Fig. 3E). These data suggest that downregulation of TMBIM6 causes degeneration of DAergic neurons in the eye of *D. melanogaster*.

### TMBIM6 deficiency exacerbates DAergic neurodegeneration in an in vivo pharmacologic model of PD

Building on the idea that TMBIM6 regulates DAergic neurodegeneration in vitro in the context of PD, the next step was to assess whether this effect also occurs in a more complex in vivo model, specifically in the *D. melanogaster* brain. To explore this, we specifically downregulated *dTmbim6* in DAergic neurons of flies using the Ple-GAL4 promoter [47]. We then developed an in vivo pharmacologic PD model by exposing *D. melanogaster* to 300 μM rotenone for 7 days (Fig. 3F). Motor ability was assessed using two approaches: a climbing assay over 7 days post-rotenone exposure and a 24-h spontaneous activity measurement. Both assays revealed a significant rotenone-induced decline in locomotor performance. si*dTmbim6* flies exhibited impaired motor behavior even under basal conditions and showed increased sensitivity to rotenone treatment, displaying more significant motor impairment than si*Cntrl* flies (Fig. 3G, H). These findings suggest that TMBIM6 deficiency exacerbates motor dysfunction induced by a PD-like neurotoxin in vivo.

Given the spontaneous decrease in locomotor activity observed in flies lacking TMBIM6 in DAergic cells, as assessed by both climbing and spontaneous activity assays, we studied the impact of TMBIM6 expression on the DAergic neuron population. To do this, we isolated the brains of si*dTmbim6* flies and stained them to visualize TH+ neurons in DAergic clusters PPL1, PPL2, PPM1/2, and PPM3 [36] (Fig. 3I). We found that si*dTmbim6* flies exhibited a basal reduction in the number of TH+ neurons compared to *siCntrl* flies. Additionally, rotenone treatment significantly decreased the number of TH+ neurons in *siCntrl* flies, but not in si*dTmbim6* flies, suggesting that the remaining TH+ neurons in si*dTmbim6* flies are resistant to rotenone-induced neurodegeneration (Fig. 3J). Therefore, we analyzed the size of DAergic neuron somas in DAergic clusters as a measure of neurodegeneration. This analysis revealed that rotenone, but not the vehicle treatment, caused a significant decrease in neuronal soma diameter in si*dTmbim6* flies compared to *siCntrl* flies (Fig. 3K). These results suggest that TMBIM6 deficiency leads to a basal reduction in TH+ neurons and that the remaining neurons exhibit resistance to rotenone treatment.

### TMBIM6 overexpression protects against PD-like cellular damage

**Fig. 4:**
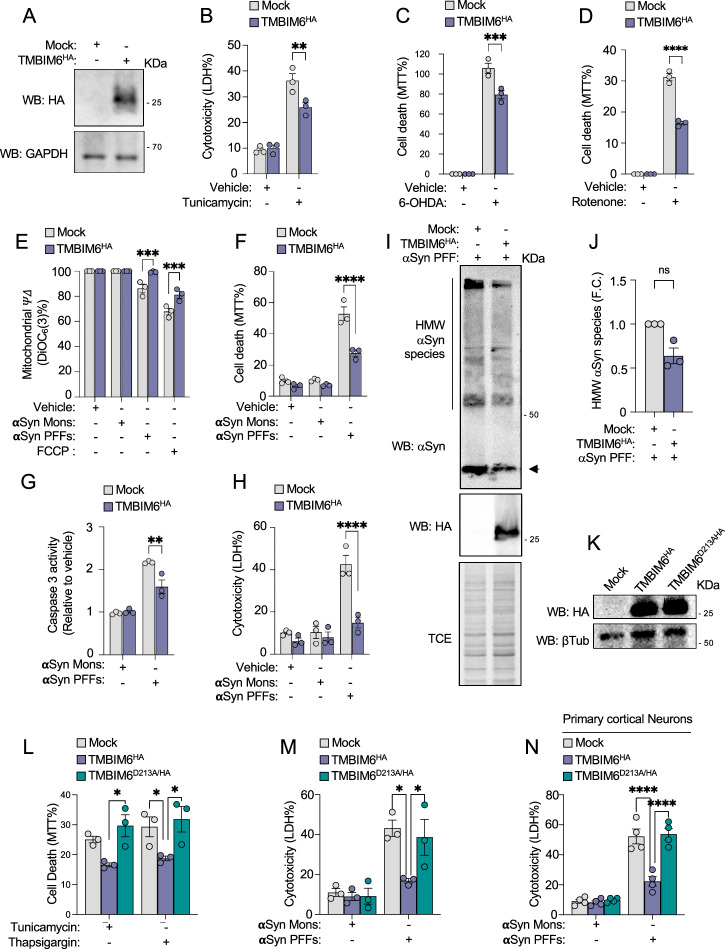
Protective effect of TMBIM6 overexpression on in vitro PD model. **A** Representative immunoblot shows a stable expression of TMBIM6^HA^ in SN4741 cells. **B** Cytotoxicity assay shows the cell death induced by Tunicamycin in SN4741 TMBIM6^HA^ cells after 24 h. **C**, **D** MTT assay shows cell death induced by 6-OHDA or rotenone in SN4741 TMBIM6^HA^ cells after 24 h. Results are expressed as % of MTT. **E** Retention of DiOC6(3) assay shows the effect of aSyn on ΔΨm in SN4741 hTMBIM6^HA^ cells after 18 h. Results are expressed as % of DiOC6(3) retention. **F** MTT assay shows cell death induced by aSyn in SN4741 TMBIM6^HA^ cells after 24 h. **G** DEVD-AMC fluorescent assay shows the effect of aSyn on Caspase-3 activity in SN4741 TMBIM6^HA^ cells after 24 h. **H** Cytotoxicity assay shows the cell death induced by aSyn in SN4741 TMBIM6^HA^ cells after 24 h. **I** Representative immunoblot of HMW aSyn species in Mock or TMBIM6^HA^ cells treated with aSyn PFFs for 24 h; TCE staining was used as a loading control. **J** Densitometric quantification of HMW aSyn bands from the experiment described in J (integrated density normalized to TCE). **K** Representative immunoblot shows expression of TMBIM6^HA^ and TMBIM6^D213A/HA^ in SN4741 cells. **L** MTT assay shows cell death induced by Tunicamycin and Thapsigargin in SN4741 Mock, TMBIM6^HA^, and TMBIM6^D213A/HA^ cells after 24 h. Results are expressed as % of MTT. **M** Cytotoxicity assay shows the cell death induced by aSyn in SN4741 TMBIM6^HA^ and TMBIM6^D213A/HA^ cells after 24 h. **N** Cytotoxicity assay shows the cell death induced by aSyn after 10 days in PCNs transfected with Mock, TMBIM6^HA^ and TMBIM6^D213A/HA^ constructs. All bars represent mean ± SEM. For experiments involving more than two groups, a two-way ANOVA followed by Tukey’s multiple comparison test was performed. Pairwise comparisons between two groups were analyzed using the Mann–Whitney U test. Statistical significance (p < 0.05) between samples is indicated in the figures as follows: *=p < 0.05; **=p < 0.01; ***=p < 0.001; ****=p < 0.0001.

After determining that the loss of TMBIM6 function increases DAergic cell death in in vitro and *D. melanogaster* PD models, we investigated whether the gain of TMBIM6 function could provide a protective effect against DAergic neuronal death in PD. To explore this idea, we generated stable TMBIM6-expressing SN4741 DAergic cells using lentiviral vectors. The human TMBIM6 was expressed with an HA tag (TMBIM6^HA^), allowing us to monitor exogenous TMBIM6 expression by immunodetection. As a control, we used an empty vector (Mock). Western blot analysis confirmed stable TMBIM6^HA^ expression, detecting a 25–27 kDa HA-tagged protein absent in Mock-transfected cells (Fig. 4A). To determine whether TMBIM6^HA^ expression was functionally active, we first tested its effect against ER stress. Cells were exposed to Tunicamycin, a well-characterized ER stress inducer, and LDH assays revealed that TMBIM6^HA^ overexpression significantly reduced Tunicamycin-induced cytotoxicity after 24 h compared to Mock cells (Fig. 4B). After confirming that TMBIM6^HA^ conferred protection against ER stress, we evaluated its role in the context of PD-related neurotoxins that do not directly induce ER stress. MTT assays showed that TMBIM6^HA^ expression significantly reduced mitochondrial-dependent cytotoxicity induced by 50 µM 6-OHDA and 25 µM rotenone after 24 h (Fig. 4C, D). Finally, we assessed whether TMBIM6^HA^ overexpression could protect against aSyn PFFs-induced toxicity. We found that TMBIM6^HA^ expression preserved mitochondrial membrane potential, maintained cell viability, prevented caspase activation, and cytotoxicity following exposure to 10 µM aSyn PFFs (Fig. 4E–H), without affecting the formation of HMW aSyn species (Fig. 4I, J). To further validate these findings, we tested TMBIM6^HA^ transient overexpression in PCNs. 48 h post-transfection (Fig. S6A), PCNs were exposed to 50 µM 6-OHDA or 25 µM rotenone, and cell death was assessed by LDH assay after 24 h. TMBIM6^HA^ expression significantly reduced neurotoxin-induced cytotoxicity compared to Mock-transfected neurons (Fig. S6B, C). Additionally, to evaluate its protective effect against aSyn PFFs toxicity, PCNs were treated with 10 µM aSyn PFFs for 7 days. TMBIM6^HA^ expression significantly reduced caspase-3 activation and cytotoxicity induced by aSyn PFFs (Fig. S6D, E). These results indicate that TMBIM6^HA^ transient overexpression reduces caspase-3-dependent neuronal death induced by PD-like neurotoxins in PCNs. Finally, the anti-apoptotic activity of TMBIM6 against ER stress depends on its ability to regulate ER Ca²⁺ levels, a function that requires an aspartate at position 213 [48]. To test if the protection of TMBIM6 against PD-stimulus is Ca^2+^-dependent, we expressed a mutant in which D213 is substituted by alanine (TMBIM6^D213A/HA^), a change previously reported in the literature [49], alongside wild-type TMBIM6^HA^ (Fig. 4K). As expected, TMBIM6^HA^ protected cells from ER stress induced by Tunicamicin and Thapsigargin, whereas the TMBIM6^D213A/HA^ failed to confer protection (Fig. 4L). When challenged with aSyn PFFs, TMBIM6^HA^ reduced cell death in both SN4741 cells and PCNs, while cells expressing TMBIM6^D213A/HA^ showed levels of death comparable to mock controls (Fig. 4M, N). Altogether, this data suggests that TMBIM6 expression does not alter aSyn aggregation but enhances neuronal survival under conditions that mimic PD in a Ca²⁺-dependent manner. This evidence provides initial support that TMBIM6 overexpression could be a valid therapeutic alternative in the context of PD.

### IRE1α pathway is active in PD patients

**Fig. 5:**
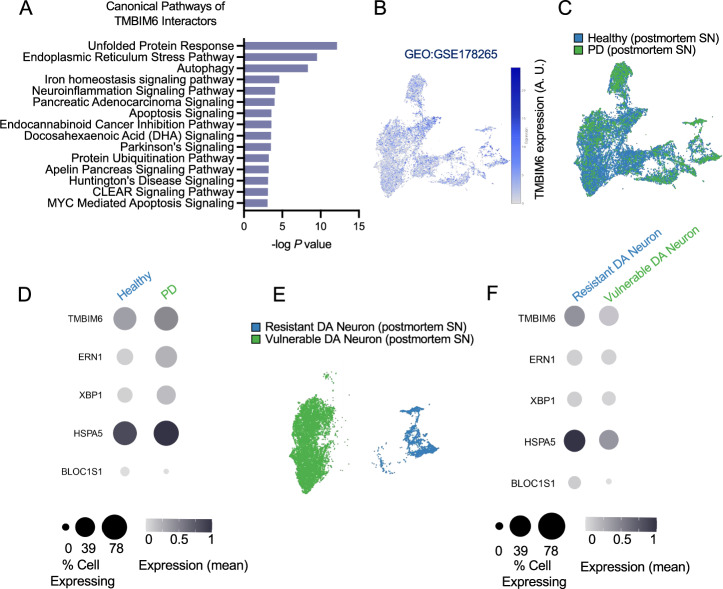
Dysregulation of TMBIM6 and UPR gene expression in the substantia nigra of PD patients. **A** An in-silico assay using Ingenuity Pathway Analysis (IPA) software shows the canonical pathways significantly associated with TMBIM6 interactors. **B**, **C** UMAP visualizations of the snRNA-seq dataset (GEO: GSE178265) from human postmortem substantia nigra. **B** shows TMBIM6 expression across all nuclei, while **C** distinguishes nuclei from healthy and PD donors. **D** Dot plot comparing the expression of *TMBIM6* and UPR-related genes (*HSPA5, ERN1, XBP1, BLOC1S1*) between healthy and PD conditions across all nuclei. **E** UMAP plot identifying resistant and vulnerable DAergic neuron populations within the dataset. **F** Dot plot comparing gene expression between resistant and vulnerable DAergic neurons within the PD cohort. For dot plots (**D**, **F**), dot size represents the percentage of cells expressing the gene, and color intensity indicates the mean expression level. Statistical significance for the differential expression shown in (**D**, **F**) was determined using the Model-based Analysis of Single-cell Transcriptomics (MAST) test. Full statistical details, including FDR-adjusted p-values, are provided in Supplementary Fig. 7F.

Previously, we demonstrated that TMBIM6 protects against cell death in PD models (Figs. 2–4 and S6); however, the cellular mechanism by which TMBIM6 confers protection against neurotoxic aSyn remains unclear. Therefore, we performed an in-silico analysis to investigate the canonical pathways associated with TMBIM6 interactors using IPA. We observed that TMBIM6 is significantly involved in UPR signaling, ER stress pathway, apoptosis, and PD signaling (Fig. 5A). Next, we examined TMBIM6 expression across 18,399 nuclei from the human SNpc using single-nucleus RNA-seq data (GEO: GSE178265), reporting overall TMBIM6 expression levels (Fig. 5B). We then focused on DAergic neurons and performed comparative co-expression analyses in neurologically healthy versus PD samples (Fig. 5C). We tested four UPR-related genes, *ERN1* (IRE1a), *XBP1*, *HSPA5* (BiP), and *BLOC1S1*, selected for their roles in ER stress responses. This analysis revealed a significant disruption in the co-expression of *TMBIM6* with key UPR effectors, including *HSPA5*, *ERN1*, and *XBP1*, in the overall cell population from PD patients compared to healthy controls, whereas *BLOC1S1* showed only a minor, non-significant change (Fig. 5D and Fig. S7A–C). We quantified these effects as Δ correlation (rPD − rHealthy) (Fig. S5D). In addition, analysis of an independent postmortem cohort showed an overall increase in TMBIM6 protein levels in bulk SNpc samples from PD patients (Fig. 1E, F). To pinpoint the cellular origin of this dysregulation, we performed a targeted differential expression analysis on DAergic neuron subtypes, vulnerable *SOX6*^*+*^*/AGTR1*^*+*^ DAergic neurons and resistant *CALB1*^*+*^*/GEM*^*+*^*, CALB1*^*+*^*/TRHR*^*+*^*, CALB1*^*+*^*/RBP4*^*+*^ DAergic neurons (Fig. 5E). Strikingly, this analysis revealed that *TMBIM6* mRNA levels were significantly reduced specifically within the population of DAergic neurons identified as vulnerable to degeneration (*SOX6*^*+*^*/AGTR1*^*+*^*)*, when compared to the more resistant DAergic neuron population (*CALB1*^*+*^*/GEM*^*+*^*, CALB1*^*+*^*/TRHR*^*+*^*, CALB1*^*+*^*/RBP4*^*+*^*)*. (Fig. 5F). Altogether, these results indicate a selective disruption of *TMBIM6* co-expression with key UPR effectors in PD and point to loss of *TMBIM6* in vulnerable DAergic neurons as a likely contributor to the condition-specific breakdown of stress-response coordination involving *HSPA5*, *ERN1* and *XBP1*.

### TMBIM6 regulates IRE1a activation induced by aSyn

**Fig. 6:**
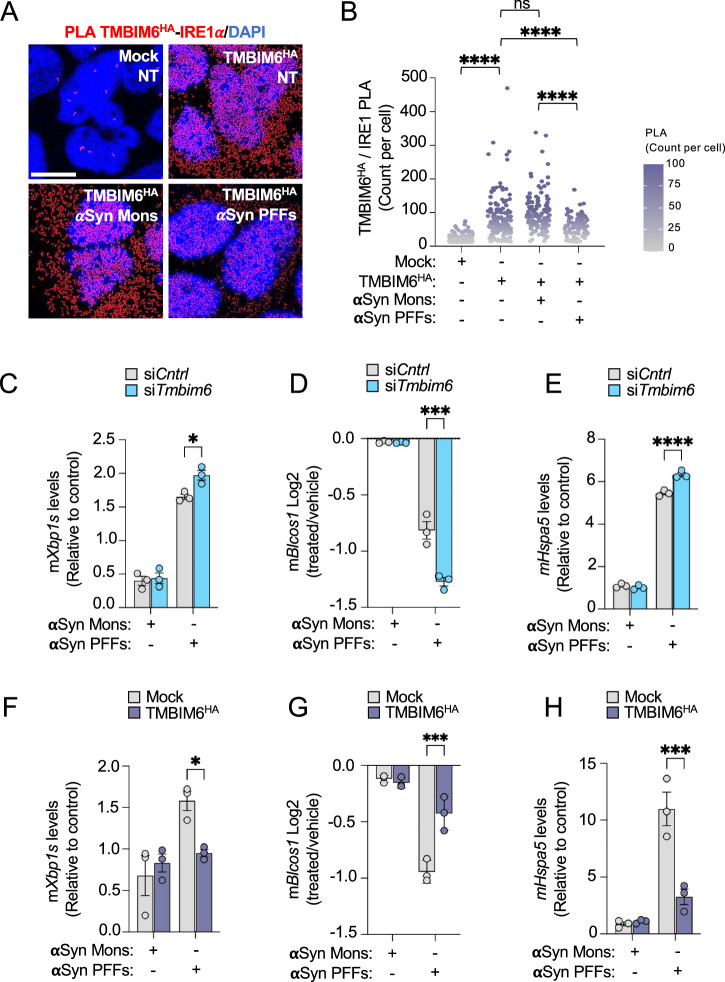
Modulation of IRE1a activity by TMBIM6 upon aSyn exposure. **A** Representative images show red fluorescent dots of PLA assay to TMBIM6^HA^/IRE1a in stable SN4741 TMBIM6^HA^ cells exposed to aSyn. **B** Quantification of PLA dots per cell. Kruskal-Wallis followed by Dunn’s multiple comparison test. **C** In SN4741 siRNA-*mTMBIM6* cells, an RT-qPCR assay shows the effect of aSyn on mRNA levels of mouse XBP1s (*mXbp1s*), **D** mouse BLOCS1 *(mBlocs1)*, and **E** mouse BIP *(mBip)*. **F** In SN4741 TMBIM6^HA^ cells, RT-qPCR assay shows the effect of aSyn in *mXbp1s*, **G** *mBlocs1*, and **H** *mBip*, mRNA levels. Results are expressed as fold change, and bars represent mean ± SEM. All bars represent mean ± SEM. In (**B**), a Kruskal–Wallis test was performed followed by Dunn’s multiple comparison test. For experiments involving more than two groups, a two-way ANOVA followed by Tukey’s multiple comparison test was performed (**C**–**H**). Statistical significance (p < 0.05) between samples is indicated in the figures as follows: *=p < 0.05; ***=p < 0.001; ****=p < 0.0001.

To explore the role of IRE1a and TMBIM6 in cell death within a PD-like context, we performed a PLA assay to evaluate their proximity in SN4741 cells under aSyn PFF overload or its absence. Interestingly, we observed that TMBIM6^HA^-IRE1a coupling is disrupted by aSyn PFFs but not by aSyn Mons after 18 h (Fig. 6A, B). Similarly, the TMBIM6^D213A/HA^ mutant, which lacks Ca^2+^-channel activity, behaved indistinguishably from TMBIM6^HA^ (Fig. S8A, B). This result suggests that TMBIM6 interacts with IRE1a in neurons under basal conditions, but when neurons are exposed to aSyn PFFs, it causes TMBIM6 to decouple from IRE1a, a process that occurs independently of its Ca^2+^-channel function. We hypothesized that this decoupling enables the activation of IRE1a necessary for the primary response to aSyn toxic species. A study in a PD *D. melanogaster* model showed that the IRE1a pathway mediates aSyn-induced cell death [13]. Since TMBIM6 is a canonical inhibitor of IRE1a, we hypothesized that TMBIM6 prevents cell death in PD models by modulating IRE1a. To explore this idea, we tested IRE1a activity induced by aSyn in SN4741 DAergic cells within a PD-like context, monitoring changes in the expression of reporter genes for IRE1a activity by RT-qPCR. We observed that aSyn PFFs, but not aSyn Mons or vehicle, increase *mXbp1s* mRNA expression, indicating IRE1a RNase activation (Fig. 6C). Additionally, we observed that TMBIM6 downregulation significantly increases *mXbp1s* mRNA expression and *mBlocs1* RNA decay induced by aSyn PFFs but not by aSyn Mons, indicating increased IRE1 activity (Fig. 6C, D). Moreover, we observed that the downregulation of *mTmbim6* significantly increases *mHspa5* induced by aSyn PFFs but not by aSyn Mons (Fig. 6E). These results demonstrate that neurotoxic aSyn increases IRE1a activity, inducing ER stress, and that TMBIM6 regulates IRE1a activity in DAergic cells.

Given that TMBIM6 depletion enhances IRE1a activation, we next employed the inverse strategy by inducing TMBIM6^HA^ overexpression and then assessing its effect on IRE1a activation induced by aSyn PFFs. We observed that TMBIM6^HA^ expression significantly reduced *mXbp1s* levels, prevented *mBlocs1* decay, and decreased *mHspa5* expression induced by aSyn PFFs (Fig. 6F–H). These results indicate that TMBIM6 overexpression inhibits IRE1a RNase activity and mitigates ER stress in DAergic neurons. Previously, the TMBIM6 C-terminus was implicated in IRE1 binding and repression [19]. Following that evidence, we tested a peptide that mimics the TMBIM6 C-terminus (TMBIM6^C-term^), using a scrambled sequence as a control (Fig. S9A–C). Both peptides reduced the PLA signal between TMBIM6 and IRE1a, but the TMBIM6^C-term^ produced a significantly larger decrease than the scrambled peptide (Scramble) (Fig. S9D, E). Despite this disruption of the TMBIM6^HA^/IRE1 interaction, treatment with TMBIM6^C-term^ peptide did not alter aSyn PFF–induced cell death (Fig. S9F), which may reflect prior aSyn PFF-mediated loss of the TMBIM6/IRE1a complex or suboptimal TMBIM6^C-term^ peptide dosing/exposure. Altogether, this set of data demonstrates that IRE1a is activated in response to aSyn PFFs, as evidenced by the dissociation of the TMBIM6^HA^-IRE1a complex observed in PLA assays and the subsequent increase in IRE1a activity. Moreover, our results indicate that TMBIM6 modulates IRE1a activation, suggesting a regulatory role for TMBIM6 in ER stress and neurodegeneration in a PD context.

### IRE1a promotes aSyn-evoked apoptosis in DAergic cells lacking TMBIM6

**Fig. 7:**
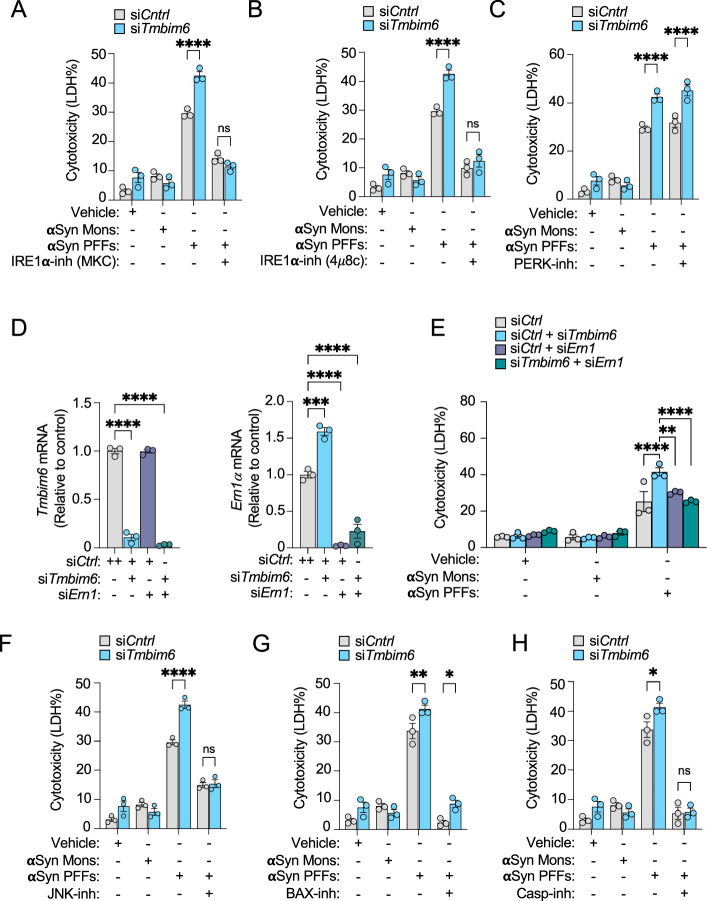
The effect of TMBIM6 modulation over the IRE1a pathway in SN4741 cell death induced by aSyn. **A**, **B** Cytotoxicity assay shows the effect of IRE1a inhibition using MKC or 4*μ*8c on cell death induced by aSyn in *Tmbim6* KD cells after 24 h. Results are expressed as % of LDH release. **C** Cytotoxicity assay shows the effect of PERK inhibitor on cell death induced by aSyn in *mTmbim6* KD cells after 24 h. **D** A RT-qPCR assay shows effective double knockdown of both *mTmbim6* (left panel) and *mIre1*a (right panel) in SN4741 cells. Results are expressed as fold change, and bars represent mean ± SEM. One way ANOVA, Dunnett´s multiple comparation test. **E** Cytotoxicity assay shows downregulation of *mIRE1a* over cell death induced by aSyn in *mTmbim6* KD cells after 24 h. Results are expressed as % of LDH release. **F** Cytotoxicity assay shows the JNK inhibitor AS60125 over cell death induced by aSyn in mTmbim6 KD cells after 24 h. **G** Cytotoxicity assay shows the BAX inhibitor BAI-1 over cell death induced by aSyn in mTmbim6 KD cells after 24 h. **H** Cytotoxicity assay shows the pan-caspase inhibitor ZVAD-FMK (casp-inh) over cell death induced by aSyn in mTmbim6 KD cells after 24 h. All bars represent mean ± SEM. In (**D**), a one-way ANOVA followed by Dunnett’s multiple comparison test was performed, whereas in (**E**, **F**, **G**, **H**), a two-way ANOVA with Tukey’s multiple comparisons test was conducted. Statistical significance (p < 0.05) between samples is indicated in the figures as follows: *=p < 0.05; **=p < 0.01; ***=p < 0.001; ****=p < 0.0001.

Because we had demonstrated that TMBIM6 inhibits IRE1a activation, we developed a model to clarify whether it is involved in cell death induced by toxic aSyn. We used SN4741 DAergic cells transfected with si*Tmbim6* and pharmacologically inhibited IRE1a to assess its role in the cytotoxicity induced by aSyn PFFs after 24 h. The LDH release assay revealed that si*Tmbim6*-transfected cells were more vulnerable to cytotoxicity induced by aSyn PFFs but not by vehicle or aSyn Mons compared to control cells. However, we observed that IRE1a inhibition with 10 µM MKC [50] or 10 µM 4µ8c [51] significantly reduced cytotoxicity induced by aSyn PFFs (Fig. 7A, B). Next, we assessed whether the regulation of DAergic cell death induced by aSyn PFFs is restricted to IRE1a or if it also involves other UPR sensors, such as PERK. To address this, we inhibited PERK using a small-compound inhibitor PERK (PERKi) [52]; however, we did not observe significant effects on cytotoxicity induced by aSyn PFFs in either control or si*Tmbim6*-transfected cells (Fig. 7C). These results suggest that aSyn PFFs activate the ER/UPR sensor IRE1a, promoting cell death. To confirm that IRE1a inhibition reverses cell death induced by aSyn PFFs, we knocked down the expression of *Ern1* (IRE1a) using siRNA in SN4741 DAergic cells transfected with si*Tmbim6*. We confirmed the successful double knockdown by RT-qPCR (Fig. 7D), where we also observed that *Tmbim6* downregulation significantly upregulated *Ire1*a mRNA levels **(**Fig. 7D, right panel). This result further supports the idea that TMBIM6 negatively regulates IRE1a. Next, we exposed the double knockdown cells to aSyn PFFs for 24 h, and cytotoxicity was measured via LDH assay. We found that genetic suppression of *Ire1a* significantly mitigated cytotoxicity induced by aSyn PFFs in the absence of *Tmbim6* (Fig. 7E). Thus, our data show that genetic inhibition of IRE1a reverses the cytotoxic effects in si*Tmbim6*-transfected DAergic cells exposed to aSyn PFFs, reinforcing the role of TMBIM6 as a repressor of IRE1a-mediated cytotoxicity. Overall, our findings demonstrate that both genetic and pharmacological inhibition of IRE1a markedly rescues aSyn-evoked cytotoxicity. This evidence supports the idea that TMBIM6 acts upstream of IRE1a in regulating the response to aSyn PFFs. Importantly, our data also place TMBIM6 downstream of aSyn PFFs. In this context, IRE1a may function as a cell death mediator in the absence of TMBIM6.

Next, we explored the downstream pathway of IRE1a involved in cell death induced by toxic aSyn species in PD models. Given that IRE1a/JNK signaling is well established [53, 54], we tested whether JNK inhibition with AS601245 [55] could rescue SN4741 DAergic cells under IRE1a hyperactivation due to TMBIM6 deficiency. JNK inhibition significantly reduced aSyn PFF-induced cytotoxicity (Fig. 7F), supporting its role in this process. Additionally, as JNK phosphorylates BCL2, leading to BAX activation [56], we inhibited BAX with the small compound BAI-1 (BAX-inh) [57]. LDH assays showed that BAX inhibition significantly reversed aSyn PFF-induced cytotoxicity (Fig. 7G), suggesting that aSyn PFFs-induced cytotoxicity requires BAX activation. To determine whether this cascade ultimately leads to apoptosis, we tested the role of caspase-3 activation. Using the pan-caspase inhibitor ZVAD-FMK (Casp-inh), we observed that caspase inhibition significantly rescued cells from aSyn PFF-induced cytotoxicity (Fig. 7H). Together, these data suggest that aSyn PFFs induce IRE1a activation. In the absence of TMBIM6, IRE1a triggers a downstream pathway that activates its RNAse activity and JNK, which in turn activates BAX and caspase-3, leading to apoptosis in DAergic neurons.

### Adeno-associated viral vector AAV-TMBIM6^HA^ prevents neurotoxicity in a PD preclinical model

Considering that TMBIM6 prevents apoptosis induced by PD neurotoxins in in vitro models of PD (Fig. 4), we developed an AAV that enables the expression of TMBIM6 with a HA tag under the control of CMV promoter and a GFP under the control of the IRES sequence (AAV-TMBIM6^HA/GFP^), or an empty vector expressing only GFP as a control (AAV-Mock^GFP^). This approach aimed to assess TMBIM6 as a potential therapeutic target in PD. To validate the functionality of the AAV-TMBIM6^HA/GFP^ vector before its use in rodents, we tested its effect on PCNs in vitro. After 96 h of transduction, GFP expression confirmed effective transduction and Western blot detection of the HA tag verified TMBIM6^HA^ expression (Fig. S10A, B). Next, we assessed whether AAV-mediated TMBIM6^HA^ overexpression replicates the neuroprotective effects previously observed with transient transfection (Fig. S6). PCNs transduced with AAV-TMBIM6^HA/GFP^ exhibited reduced cytotoxicity upon 6-OHDA and aSyn PFFs exposure (Fig. S10C, D) and diminished caspase-3 activation in response to aSyn PFF compared to AAV-Mock^GFP^ controls (Fig. S10E). These results, obtained in primary neurons, confirm the functional effectiveness of the AAV-TMBIM6^HA/GFP^, supporting its use in subsequent in vivo experiments.

**Fig. 8:**
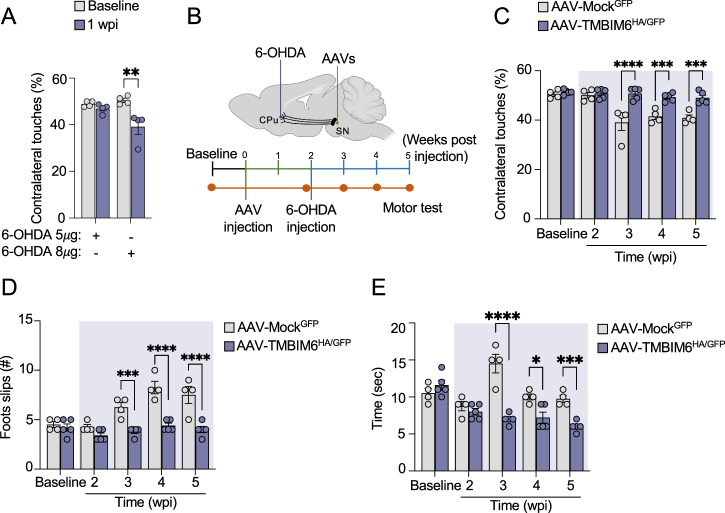
Impact of the AAV-TMBIM6^HA/GFP^ expression on in vivo PD model. **A** Effect of two 6-OHDA doses on motor performance of mice, as a pharmacologic in vivo PD model (n = 4 per condition). Results are expressed as a percentage of contralateral forelimb use. **B** Timeline of in vivo transduction of the AAV-TMBIM6^HA/GFP^ and AAV-Mock^GFP^ in SN and motor performance measurements in mice wild-type lesioned with 6-OHDA in CPu. **C** Cylinder test shows the effect of AAV-TMBIM6^HA/GFP^ and AAV-Mock^GFP^ expression in SN over forelimb use after 2-, 3-, 4-, and 5-weeks post-injection (wpi). The colored area shows treatment with 6-OHDA injuries in the CPu. Results are expressed as a percentage of contralateral forelimb use (n_Mockl _= 4 and n_TMBIM6_ = 5). **D** Beam test shows the effect of the AAV-TMBIM6^HA/GFP^ and AAV-Mock^GFP^ expression in SN on balance and coordination after 2, 3, 4, and 5 weeks after injection. The colored area shows treatment with 6-OHDA injury in the CPu. Results are expressed as the number of paws slips (n_Mockl _= 4 and n_TMBIM6_ = 5). **E** Effect of the AAV-TMBIM6^HA/GFP^ and AAV-Mock^GFP^ expression over time, animals used to complete the Beam test during 2-, 3-, 4-, and 5-wpi. The colored area shows treatment with 6-OHDA injury in the CPu. Results are expressed as the time in seconds to complete the test (n_Mockl _= 4 and n_TMBIM6_ = 5). All bars represent mean ± SEM. In all tests, two-way ANOVA followed by Tukey’s multiple comparison test was performed. Statistical significance (p < 0.05) between samples is indicated in the figures as follows: *=p < 0.05; **=p < 0.01; ***=p < 0.001; ****=p < 0.0001.

The next step was to determine the potential protection provided by AAV-TMBIM6^HA/GFP^ in a preclinical idiopathic PD model. First, we tested two doses of 6-OHDA to generate the PD model. We delivered a low 6-OHDA (5 µg) or a high 6-OHDA dose (8 µg) into the CPu of adult wild-type females (3 months old). Motor performance was monitored using a cylinder test 2 weeks post-injection (wpi) to assess PD-like neuronal damage [58]. We observed that only a high dose of 6-OHDA caused significant motor impairment (Fig. 8A). Thus, we proceeded with this 6-OHDA dose to generate an idiopathic PD model in mice. Next, we induced the expression of TMBIM6^HA^ via AAV vectors in the bilateral SN adult wild-type female mice (3 months old) using stereotaxic surgery. AAV-Mock^GFP^ was used as a control. After 2 wpi, we injured the right CPu area with a high 6-OHDA dose to model idiopathic PD (Fig. 8B). We evaluated mice’s motor performance over time as an indicator of PD-like neuronal damage **(**Fig. 8B**)**. We found that AAV-Mock^GFP^ mice showed a significant reduction in left forelimb use during exploration due to the 6-OHDA lesion, in contrast to AAV-TMBIM6^HA/GFP^ mice (Fig. 8C). Interestingly, AAV-TMBIM6^HA/GFP^ mice showed a significant reduction in the number of errors and time to complete the Beam Test compared to AAV-Mock^GFP^ mice (Fig. 8D, E). These data provide the first evidence that human TMBIM6 expression prevents motor impairment in a preclinical model of idiopathic PD.

## Discussion

PD is the second most frequent multifactorial neurodegenerative disorder in aging people [1]. PD is a devastating neurodegenerative disease characterized by progressive impairment of voluntary motor control caused by progressive loss of midbrain SNpc DAergic neurons [5]. The early and cardinal event in PD pathology is the toxic accumulation and aggregation of misfolded aSyn in the cytoplasm of DAergic presynaptic neurons, which precedes SNpc DAergic neuron loss, neurodegeneration, and motor deficits [5, 59]. PD has no known cure, posing a significant public health challenge [60]. Therefore, the search for novel therapeutic strategies for the management and prevention of PD is urgently needed. aSyn aggregation and accumulation trigger several changes in proteostasis, resulting in ER stress, UPR activation, mitochondrial dysfunction, and other severe cellular stress processes that culminate in DAergic neuron death [9, 61, 62]. In this context, TMBIM6 is a highly conserved ancestral anti-apoptotic protein that inhibits ER stress-induced cell death BAX-mediated apoptosis [63]. However, whether TMBIM6 could be a novel therapeutic factor for PD has not been investigated. Here, we propose that TMBIM6 plays a protective role in PD and present evidence supporting the hypothesis that TMBIM6 actively represses DAergic neuron death.

For the first time, using bioinformatics analysis and RT-qPCR assays, we demonstrate that *TMBIM6* is expressed in the CNS and is highly expressed in the midbrain, which is an area enriched in DAergic neurons located in the SNpc [41]. Therefore, we hypothesize that TMBIM6 has a significant neurobiological role in DAergic neurons. We present experimental evidence indicating an acute increase in *TMBIM6* expression in response to PD-stimuli. We speculate that this acute increase is a response to elevated IRE1α activity and the production of reactive oxygen species induced by PD-stimuli [64]. Subsequently, in our model of PD *TMBIM6* expression declines over time, allowing an increase in the expression of the pro-apoptotic mitochondrial factor BAX, leading to neuronal death. BAX-mediated cell death is a well-established mechanism identified in PD pathology [65]. Additionally, we observed in samples from a cohort of PD patients that TMBIM6 expression increases in the SNpc but decreases specifically in vulnerable DAergic neurons, suggesting that in the advanced stage of PD, the increase in TMBIM6 may occur in other cellular components, while the reduction of TMBIM6 specifically in vulnerable DAergic neurons could predispose them to BAX-mediated cell death.

Given that PD affects other tissues in the body [66], we also evaluated *TMBIM6* expression in PBMCs from PD patients. However, we did not find changes in TMBIM6 expression in this peripheral tissue from living PD patients. This could be explained by the fact that the analyzed sample only included subjects in stages 1-4 on the H&Y scale but not in stage 5, where neurodegeneration is more severe. Furthermore, the sample size may have been too small. Therefore, other strategies, such as measuring the protein levels of TMBIM6 protein in PBMCs or including a cohort of patients in stage 5, may provide more insight.

Based on these observations, we conclude that although TMBIM6 is a stress-sensitive response element to early PD-associated neuron damage that ensures DAergic neuron survival, we hypothesize that cell death signals surpass the survival signals over time in PD, leading to TMBIM6 downregulation and neuron cell death. The in vivo *D. melanogaster* model supports this concept: TMBIM6 deficiency leads to neurodegeneration in eye neurons [67, 68]. Interestingly, loss of TMBIM6 function in DAergic neurons exacerbates PD-like cell damage. These findings underscore the importance of TMBIM6 in maintaining neuronal survival.

Our data support a dual role for TMBIM6: under basal conditions, it is required to maintain DAergic neuron structural integrity and survival. Indeed, a single-nucleus RNA-seq re-analysis of human SNpc reveals that the DAergic subpopulation identified as vulnerable in PD displays significantly reduced TMBIM6 levels, supporting the idea that loss of TMBIM6 contributes to baseline neuronal susceptibility. Upon exposure to PD-relevant insults (e.g., aSyn PFFs, 6-OHDA, rotenone), TMBIM6 provides additional protection by limiting IRE1a-dependent pro-apoptotic signaling. In part, this protection reflects a direct physical interaction between TMBIM6 and IRE1a that represses IRE1a RNAse activity; importantly, aSyn PFFs disrupt this TMBIM6–IRE1a complex, placing aSyn PFFs upstream of IRE1a activation. Furthermore, TMBIM6 exerts a second, complementary effect through regulation of ER luminal Ca²⁺, which indirectly reduces mitochondrial outer membrane permeabilization and thereby limits BAX-mediated apoptosis. Consistent with this two-tiered model, a TMBIM6^D213A/HA^ mutant that abrogates ER Ca²⁺ regulation loses the ability to protect against ER-stress inducers and shows reduced anti-apoptotic efficacy in PD models, indicating that Ca²⁺ regulation is necessary to achieve full neuroprotection. In d*Tmbim6* knockdown flies, the lack of further cell loss after rotenone most likely reflects early degeneration of the most vulnerable TH⁺ neurons, which reduces the measurable dynamic range for additional depletion. In line with these observations, single-cell transcriptomic re-analysis of postmortem SNpc from PD patients shows a selective reduction of TMBIM6 in the DAergic neuronal subpopulation identified as vulnerable to degeneration, strengthening the translational relevance of our model. Crucially, however, complementary endpoints—reduced soma size, histological signs of subcellular degeneration, and worsened motor behavior—demonstrate that rotenone nevertheless exacerbates neuronal pathology in the d*Tmbim6* knockdown background. Thus, TMBIM6 is critical both for basal DAergic neuron homeostasis and for resilience to toxin-induced pathology, and a multiparametric readout is required to capture the full spectrum of degeneration.

aSyn aggregation and accumulation in DAergic neurons disrupt the cellular machinery essential for proper proteostasis. Misfolded aSyn overload can trigger ER stress and UPR [69], including IRE1a pathway activation, which initially promotes cellular survival but leads to cell death if chronically activated [70, 71]. Given that TMBIM6 is a known inhibitor of the IRE1a pathway [16, 17, 22, 64, 72], we hypothesized that TMBIM6 promotes neuroprotection through IRE1a pathway modulation in PD models. We demonstrated that aSyn induces *XBP1* mRNA splicing and *BLOCS1* downregulation, indicating IRE1a pathway activation. The absence of TMBIM6 exacerbates aSyn-evoked IRE1a pathway activation. Interestingly, TMBIM6 overexpression completely mitigates IRE1a pathway activation induced by aSyn. Using DAergic cells lacking TMBIM6, we demonstrated that aSyn-evoked IRE1a activation-mediated cell death is prevented by IRE1a inhibition. Together, our data show that cell death regulators downstream of the IRE1a pathway, such as JNK and BAX, are involved in aSyn-evoked IRE1a pathway activation. Collectively, our findings suggest that TMBIM6 overexpression inhibits IRE1a activation.

Finally, our research shows that TMBIM6 could be a neuroprotective factor in PD-like neurodegeneration. Here, we assessed the effect of TMBIM6 overexpression on cell death in PD models for the first time. Using lentivirus vectors, we generated a stable DAergic SN4741 cell line that overexpressed human TMBIM6. This overexpression resulted in the preservation of mitochondrial membrane potential, decreased caspase-3 activity, and prevention of aSyn-evoked DAergic cell death. This finding opened the possibility of studying TMBIM6 as a novel neuroprotective factor. Building on this, we induced TMBIM6 expression in the SN of a PD mouse model via AAV vectors. We present strong evidence that TMBIM6 expression prevents motor impairment in an in vivo PD model, as evidenced by improved motor skills in mice overexpressing TMBIM6. This novel finding leads us to conclude that TMBIM6 expression provides neuroprotection in PD. However, because these in vivo data derive from a 6-OHDA oxidative-stress model, future experiments in aSyn–based models (e.g., AAV–aSyn or PFF inoculation) are needed to explore yet unknown aspects of this pathway, such as the effect of TMBIM6 expression on mitochondrial homeostasis, Ca^2+^ flux, neuroplasticity, and aSyn aggregation or propagation. Our work is the first to present evidence of the neuroprotective effect of TMBIM6 in PD and contributes to new findings about the role of TMBIM6 in neuronal cells. Our results also suggest TMBIM6 as a potential therapeutic target for PD and other neurodegenerative disorders.

## Conclusions

Finally, we can conclude that TMBIM6 functions as a response element against PD-stimuli, ensuring DAergic neuron survival. However, in the advanced stages of PD, cellular stress exceeds the threshold of cell survival, and TMBIM6 expression decreases in vulnerable DAergic neurons, favoring chronic activation of the IRE1a pathway, which predisposes DAergic neurons to BAX-mediated cell death. Overexpression of TMBIM6 in DAergic neurons promotes neuroprotection through IRE1a pathway inhibition, resulting in (i) preservation of mitochondrial membrane potential, (ii) decreased caspase-3 activity, and (iii) prevention of aSyn-evoked DAergic cell death. Moreover, TMBIM6 overexpression prevents motor impairment in a preclinical PD model.

## Supplementary information

Supplementary Figure Legends

Supplementary Figures

Supplementary Statistics Table

## Data Availability

The datasets used and/or analyzed during the current study are available from the corresponding author upon reasonable request.
