# Supplementary material for: TMBIM6 enhances dopaminergic neuron survival by modulating the IRE1a pathway in Parkinson’s disease
Source: Cell Death Dis. 2026 Apr 3;17(1):385. doi: 10.1038/s41419-025-08391-5 (PMC13061952; doi:10.1038/s41419-025-08391-5)
Supplement: Supplementary file 1 — Supplementary Figure Legends [file 41419_2025_8391_MOESM1_ESM.docx]

**Figure Supplementary 1. Primers and *D. melanogaster* Stocks. A)** Table of Mouse Primers sequences. **B)** Table of Human Primers sequences. **C)** *D. melanogaster* Primers Sequences. **D)** *D. melanogaster* Stocks.

**Figure Supplementary 2. Regulation of BCL2 and BAX expression in PD. A)** *Bcl2* mRNA level at longtime in PCNs exposed to aSyn for 96 hours. **B)** mRNA expression of *BAX* in PCNs exposed to aSyn by 96 hours. Results are expressed as fold change, and bars represent mean ± . In all tests, two-way ANOVA followed by Tukey's multiple comparison test was performed.

**Figure Supplementary 3. TMBIM6 Expression in Postmortem PD Substantia Nigra Samples. A)** Clinical characterization of the postmortem cohort. HC, healthy control. **B)** Sample list for Gel 1 and Gel 2 with patient ID, age, sex, and disease progression (months). **C)** Scatter plot showing the relationship between PD progression (months) and measured TMBIM6 protein levels in substantia nigra extracts. **D)** Full western-blot films used for the quantification presented in Figure 1E. Red asterisks mark lanes excluded from analysis due to transfer/technical issues. The lateral arrow indicates the quantified band; the purple box highlights the region selected for display and quantification in Figure 1F. Protein signals were normalized to GAPDH before analysis.

**Figure Supplementary 4.** **Regulation of TMBIM6 expression in a living PD cohort. A)** Summary table of clinical characteristics for the Chilean cohort analyzed. HC, healthy control. **B)** Sample list corresponding to panels C–D, showing patient ID, age, sex, and disease duration (years). **C)** *TMBIM6* mRNA levels measured in PBMCs from healthy controls (HC, n = 13) and PD patients (PD, n = 16). Unpaired t-test. **D)** *TMBIM6* mRNA levels stratified by sex (Male n = 14; Female n = 15). Unpaired t-test. **E)** Scatter plot showing the relationship between Hoehn & Yahr (H&Y) score and *TMBIM6* mRNA in PBMCs. **F)** Scatter plot showing the relationship between UPDRS part III score and *TMBIM6* mRNA in PBMCs. **G)** Scatter plot showing the relationship between disease duration (years since diagnosis) and *TMBIM6* mRNA in PBMCs. Correlation analyses were performed using Spearman’s rank correlation; exact r and p values are reported in the panels. In panels C–D, bars indicate mean ± SEM, and each dot represents an individual subject. Statistical tests and sample sizes are indicated in the figure panels.

**Figure Supplementary 5. Effect of the in vivo dTMBIM6 downregulation on *D. melanogaster.* A)** Fly-eye integrity scale, indicating a damage category from 1 to 5 based on an ALS model, where 5 represents an optimal development, and 1 indicates severe damage. **B)** Frequency of distribution of the flies from A and B into eye integrity score, incubated at 25ºC (n=50).

**Figure Supplementary 6. Impact of TMBIM6 expression on in vitro PD models.** **A)** Representative immunoblot shows a transient expression of TMBIM6^HA^ in PCNs after 48 hours. **B and C)** Cytotoxicity assay shows the cell death induced by 6-OHDA or rotenone in PCNs TMBIM6^HA^ cells after 24 hours. Results are expressed as % of LDH release . **D)** DEVD-AMC fluorescent assay shows Caspase-3 activity in PCNs TMBIM6^HA^ cells after 7 days of aSyn. Results are expressed as fold change of DEVD-AMC fluorescence intensity . **E)** Cytotoxicity assay shows the cell death induced by aSyn in PCXNs TMBIM6^HA^ cells after 7 days . All bars represent mean ± SEM. In all tests, two-way ANOVA followed by Tukey's multiple comparison test was performed. Statistical significance (p < 0.05) between samples is indicated in the figures as follows: *=p<0.05; **=p<0.01; ***=p<0.001; ****=p<0.0001.

**Figure Supplementary 7. Co-expression of *TMBIM6* with UPR genes in substantia nigra of PD patients.** Scatter plots showing the relationship between *TMBIM6* expression and each UPR-related gene in healthy (gray) and PD (purple) samples: **A)** *ERN1*, **B)** *HSPA5*, and **C)** *XBP1*. Each point represents a single nucleus (n = 18,399 total nuclei). Expression values are scaled per gene. Dashed lines indicate linear regression for each condition. Reported *p*-values correspond to Fisher’s Z-test, comparing the Pearson correlation coefficients between control and PD groups. **D)** Bar graph showing the change in correlation (Δ correlation = cor_PD − cor_Healthy) between *TMBIM6* and four UPR genes. Data are based on 18,399 single nuclei. Statistical significance was evaluated using Fisher’s Z-test, with p-values shown above each bar. **E)** Summary table of correlation analysis results. Columns include: Correlation in healthy controls (cor_normal), Correlation in PD (cor_parkinson), Δ correlation (delta_cor), Fisher’s Z-score, Raw and adjusted p-values, Significance designation (adjusted p < 0.05). Statistical Summary of Differential Gene Expression Analysis from Human Substantia Nigra snRNA-seq Data. **F)** These tables summarize the statistical results of a differential gene expression analysis using the MAST test on a public snRNA-seq dataset39 (GEO: GSE178265). The upper section compares all nuclei between healthy controls and PD patients (**Figure 5D**). The lower section presents a targeted comparison between vulnerable (*SOX6^+^/AGTR1^+^*) and resistant (*CALB1^+^/GEM^+^, CALB1^+^/TRHR*, and *CALB1^+^/RBP4^+^*) DAergic neuron subtypes within the PD cohort (Figure 5F). For each comparison, the table provides the log₂(Fold Change) (logFC), mean log-normalized expression, and the percentage of nuclei expressing each gene. Statistical outputs from the MAST hurdle model include the overall p-value (p_val_hurdle), along with p-values for the continuous (p_val_cont) and discrete (p_val_disc) components of the model. The hurdle p-value was adjusted for multiple comparisons using the Benjamini-Hochberg method (adj_p_val_hurdle), and an FDR-adjusted p-value < 0.05 was considered statistically significant.

**Figure Supplementary 8. TMBIM6^D213A/HA^ dissociates from IRE1a in response to aSyn PFFs: A)** Representative confocal images of the Proximity Ligation Assay (PLA) for the TMBIM6^D213A/HA^–IRE1a interaction in SN4741 cells. Cells expressing the mutant were either untreated (NT) or exposed to 10 µM αSyn PFFs for 18 hours. Red dots indicate protein-protein interaction, and nuclei are stained with DAPI (blue). Scale bar, 10 µm. **B)** Quantification of PLA dots per cell from the experiment described in **(A)**, including a comparison with cells expressing wild-type TMBIM6^HA^ to show that both constructs behave similarly. Statistical significance between the genotype or treated groups was determined by a Kruskal-Wallis test followed by Dunn's multiple comparison test. ***p < 0.001.

**Figure Supplementary 9. Effect of the TMBIM6C-term on the TMBIM6–IRE1a complex and aSyn PFF-induced cell death. A)** Schematic of TMBIM6 highlighting the C-terminal region; peptide sequences used are shown (TMBIM6^C-term^: RRRRRRRRLAMNEKDKKKEKK; scrambled control: RRRRRRRRKEKMKLKANKEDK). **B and C)** Mass spectrometry (MS) spectra confirming the identity and purity of the synthesized TMBIM6^C-term^ mimic peptide (B) and the scrambled control peptide (C). **D)** Representative Proximity Ligation Assay (PLA) images showing TMBIM6^HA^–IRE1a interactions in SN4741 cells treated with vehicle, scrambled, or TMBIM6^C-term^ peptides. Nuclei are counterstained with DAPI (green); PLA signal appears as magenta dots (arrowheads). Scale bar = 10 μm. **E)** Quantification of PLA dots per cell. Each point represents a single cell. F) SN4741 cells were treated with 10 μM scrambled or TMBIM6^C-term^ peptide together with PBS, aSyn Monomers (Mons), or aSyn PFFs for 24 h, and cytotoxicity was assessed by MTT. The graph shows mean ± SEM; each filled circle corresponds to an independent experiment performed in duplicate. Statistical significance between the aSyn PFF-treated groups was determined by a Kruskal-Wallis test followed by Dunn's multiple comparison test. Statistical significance (p<0.05) between samples is indicated in the figures as follows: *=p<0.05; ***=p<0.001.

**Figure Supplementary 10. Impact of the AAV-TMBIM6^HA/GFP^ expression on in vitro models of PD.** **A)** Representative immunoblot shows the expression of both AAV-Mock^GFP^ or AAV-TMBIM6^HA/GFP^ in PCNs after 7 days. **B)** Timeline of AAV-TMBIM6^HA/GFP^ and AAV-Mock^GFP^ expression in PCNs and its effects on cell death assays. **C)** Cytotoxicity assay shows the effect of AAV-TMBIM6^HA/GFP^ expression on cell death induced by 6-OHDA after 24 hours. Results are expressed as % of LDH release. **D)** Cytotoxicity assay shows the effect of AAV-TMBIM6^HA/GFP^ and AAV-Mock^GFP^ expression over cell death induced by aSyn after 7 days. **E)** DEVD-AMC fluorescent assay shows the effect of AAV-TMBIM6^HA/GFP^ expression on Caspase-3 activity induced by aSyn 7 days . All bars represent mean ± SEM. In all tests, two-way ANOVA followed by Tukey's multiple comparison test was performed. Statistical significance (p<0.05) between samples is indicated in the figures as follows: *=p<0.05; ***=p<0.001.

**Figure Supplementary 11. Western blot membranes. A)** PDVF membranes of immunoblot to TMBIM6 mouse antibody and b-Tubulin rabbit antibody in SN4741 cells homogenize. **B)** Nitrocellulose membrane of immunoblot to HA rabbit antibody and β-Tubulin rabbit antibody in Primary cortical neurons homogenates. **C)** Nitrocellulose membrane of immunoblot to HA rabbit antibody and β-Tubulin rabbit antibody in SN4741 cells homogenate. **D)** Nitrocellulose membrane of immunoblot to Ha rabbit antibody, GFP rabbit antibody, and β-Tubulin rabbit antibody in Primary cortical neurons homogenized.
