# Supplementary material for: TMBIM6 enhances dopaminergic neuron survival by modulating the IRE1a pathway in Parkinson’s disease
Source: Cell Death Dis. 2026 Apr 3;17(1):385. doi: 10.1038/s41419-025-08391-5 (PMC13061952; doi:10.1038/s41419-025-08391-5)
Supplement: Supplementary file 2 — Supplementary Figures [file 41419_2025_8391_MOESM2_ESM.pdf]

A

Mouse Primers Sequences.

Target	Forward sequence	Reverse sequence
mTMBIM6	5´-TTAGCAGAGCAGGGAAGCTC-3´	5`-ACTATTGCAGGCTGTTGCGA-3´
mBCL2	5´-GAACTGGGGGAGGATTGTGG-3´	5´-GCATGCTGGGGCCATATAGT-3´
mBAX	5´-TCAAGGCCCTGTGCACTAAA-3´	5´-GGAGGCCTTCCTAATGCCAAC-3´
mXBP1s	5´-TGCTGAGTCGGCAGCAGCAGGTG-3´	5´-GACTAGCAGACTCTGGGGAAG-3´
mBIP	5`-TCATCGGACGCACTTGGAA-3´	5´-CAACCACCTTGAATGGCAAGA-3´
mBLOCS1	5´-AACACCAAGCCAAGCAGAACGA-3´	5´-TCACCTCATGGTCCAGTiCTTTCTCT-3´
mRPL-19	5´-ACCGCCATATGTATCACAGCCTGTA-3´	5´-CGCTTTCGTGTCTCCTTGGTCTTA-3´
mACT	5´-TACCACCATGTACCCAGGCA-3´	5´-CTCAGGAGGAGCAATGATCTTGAT-3´

B

Human Primers Sequences.

Target	Forward sequence	Reverse sequence
hTMBIM6	5´-GAGAGGCGGGTTAGGAAGAGT-3´	5`-GCCACAAACATACAAAGGGCA-3´
h18s	5´-CCCTGCCCTTTGTACACACC-3´	5´-CGATCCGAGGGCCTCACTA-3´
hSHDA	5´-GAGGCAAGGGTTTAATACAGCA-3´	5´-CCAGTTGTCCTCCTCCATGT-3´

C

D. melanogaster Primers Sequences.

Target	Forward sequence	Reverse sequence
dTMBIM6	5´-ACGCAAAAGGAGGAGCGCAAA-3´	5´-TGCCTCCAAAATCCGTGTCA-3´
18S	5´-ATGTCGGCTCTTCCTATCA-3´	5´-TGTCTCACGACGGTCTAAA-3´

D

D. melanogaster Stocks

Fly Stock	Cat #	Description
GMR-Gal4	BL#1104	Promoter to specific expression in eye-specific cells
Elav-Gal4	BL#8765	Promoter to specific expression in whole neurons
Ple-Gal4	BL#8848	Promoter to specific expression in DAergic neurons
UAS-Trip	BL#35787	Control of small interferent expression
UAS-RNAi-tmbim6	VDRC#37108	Expression of small interferent RNA target tmbim6 under UAS

Fig. S2. Regulation of BCL2 and BAX expression in PD

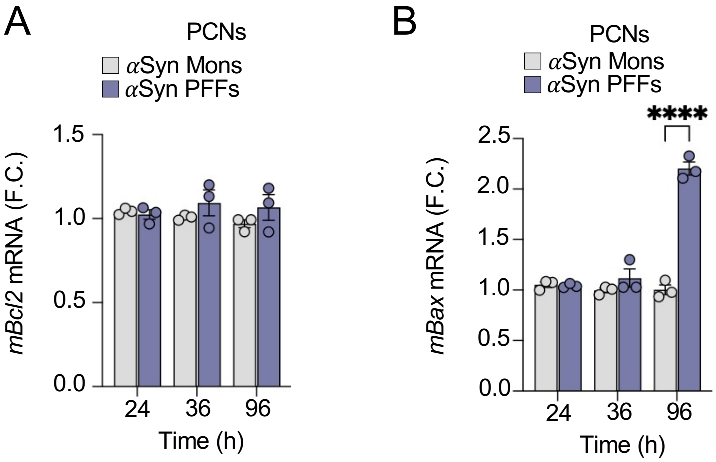

Fig. S3. TMBIM6 Expression in Postmortem Substantia Nigra (PD Satients)

A

SN post mortem	HC	PD	p
Total	10/13	9/12	-
Age	82.85	78	0.2171
Men	6	6	-
Woman	4	3	-

B

**Gel**	ID Patient	Age	Sex	Progression (months)
1	C-6203	78	M	23
1	PD-8249	79	M	27
1	C-8866*	84	M	15.5
1	PD-7958	79	M	32
1	C-4339*	86	F	37
1	PD-7291*	89	F	48
1	C-471*	81	F	12
1	PD-8175	79	F	30
1	C-342	85	M	18
1	PD-4332	79	F	36
1	C-352	75	F	14.5
1	PD-456	68	F	13
1	C-5005	92	M	17
1	Control	93	F	65
2	C-368	87	F	18.5
2	PD-478	82	M	24
2	C-460*	80	F	8
2	PD-452	77	M	8
2	C-455	79	F	7
2	PD513*	76	M	5
2	C-451	79	F	16
2	PD-480*	70	M	37
2	C-6203	78	M	23
2	PD-4849	79	M	27
2	C-8866	84	M	15.5
2	PD-7958	79	M	32
2	C-5005	92	M	17
2	Control	93	F	65

C

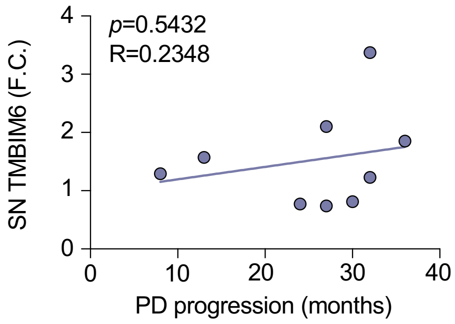

D

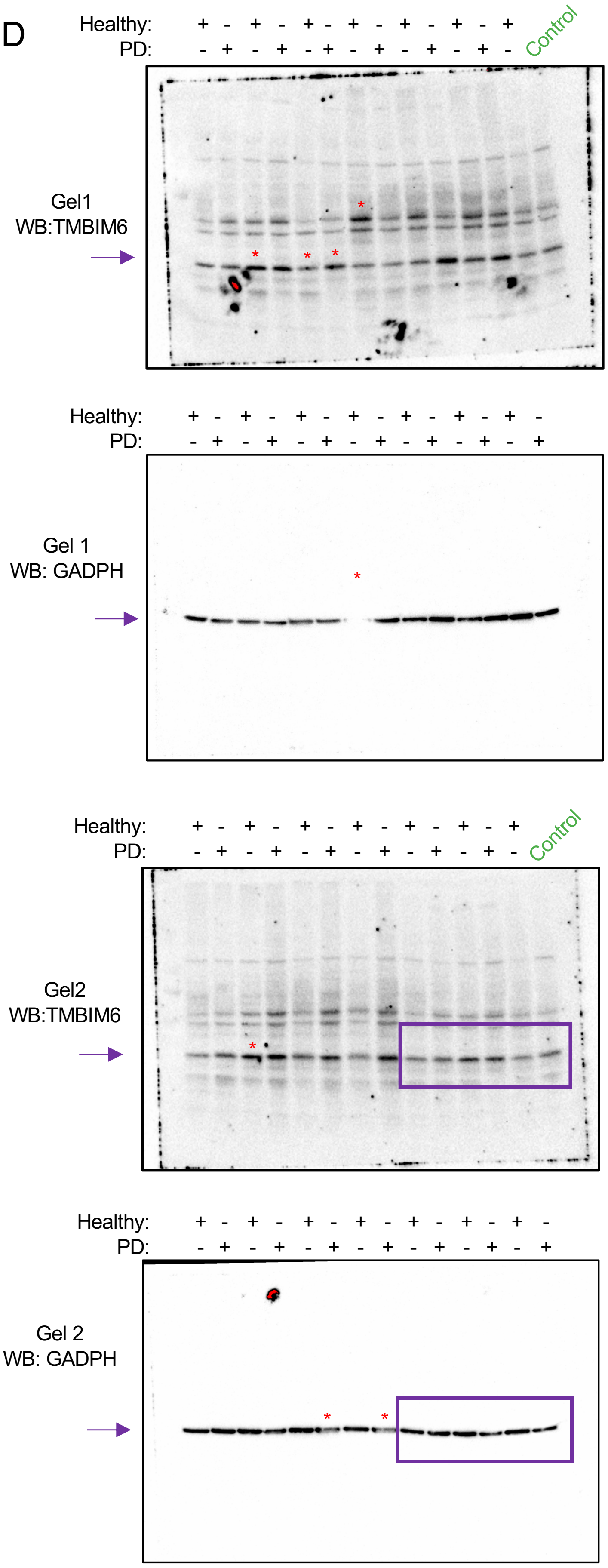

Fig. S4. Regulation of TMBIM6 expression in a living PD cohort

A

Chilean cohort - hPMBCs	HC	PD	p
Total	13	16	-
Age	67.85	70.25	0.47
Men	6	8	-
Woman	7	8	-

B

ID Patient	Age	Gender	Hoehn y Yahr Scale	UPDRS 3 Scale	Progression disease (years)
PD-11	71	M	2	31	4
PD-12	79	W	4	36	15
PD-13	60	M	3	46	5
PD-14	64	M	2	17	3
PD-16	71	W	3	26	5
PD-18	77	W	2	21	3
PD-19	70	W	3	30	29
PD-21	61	W	3	44	3
PD-22	83	W	3	56	10
PD-36	65	M	2	50	15
PD-54	78	M	2	31	6
PD-57	69	W	3	31	12
PD-87	63	M	3	37	8
PD-93	67	M	2	46	15
PD-94	64	W	2	46	15
PD-103	82	M	3	35	5

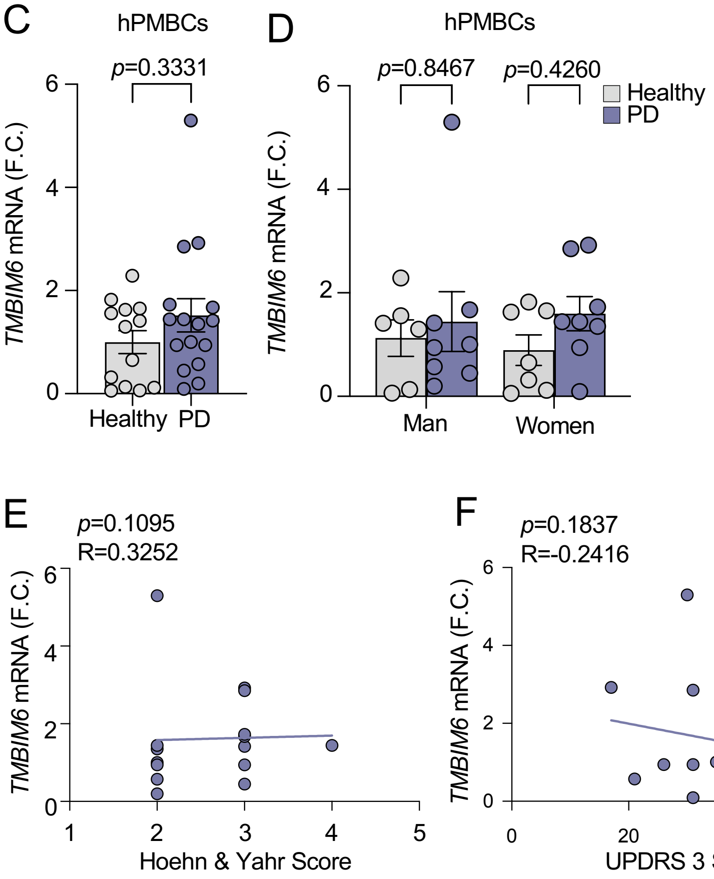

Fig. S5. Effect of the *in vivo dTMBIM6* downregulation on *D. melanogaster*

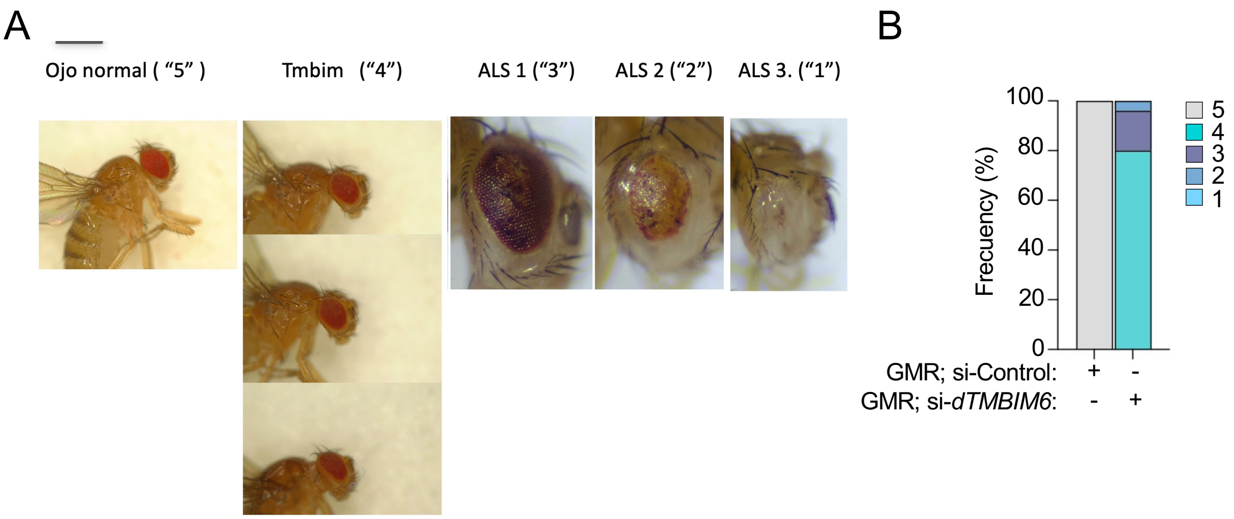

Fig. S6. Impact of TMBIM6 expression on in vitro PD models

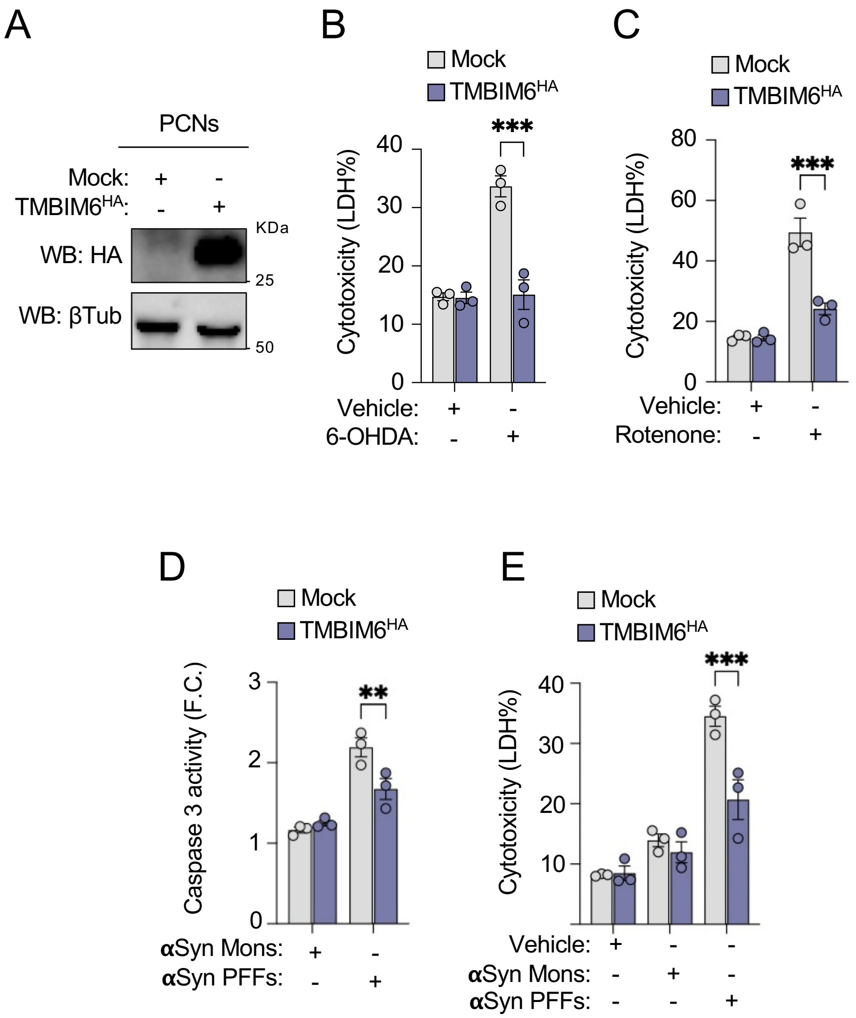

Fig. S7. Altered TMBIM6 Expression and UPR Gene Correlations in PD SN

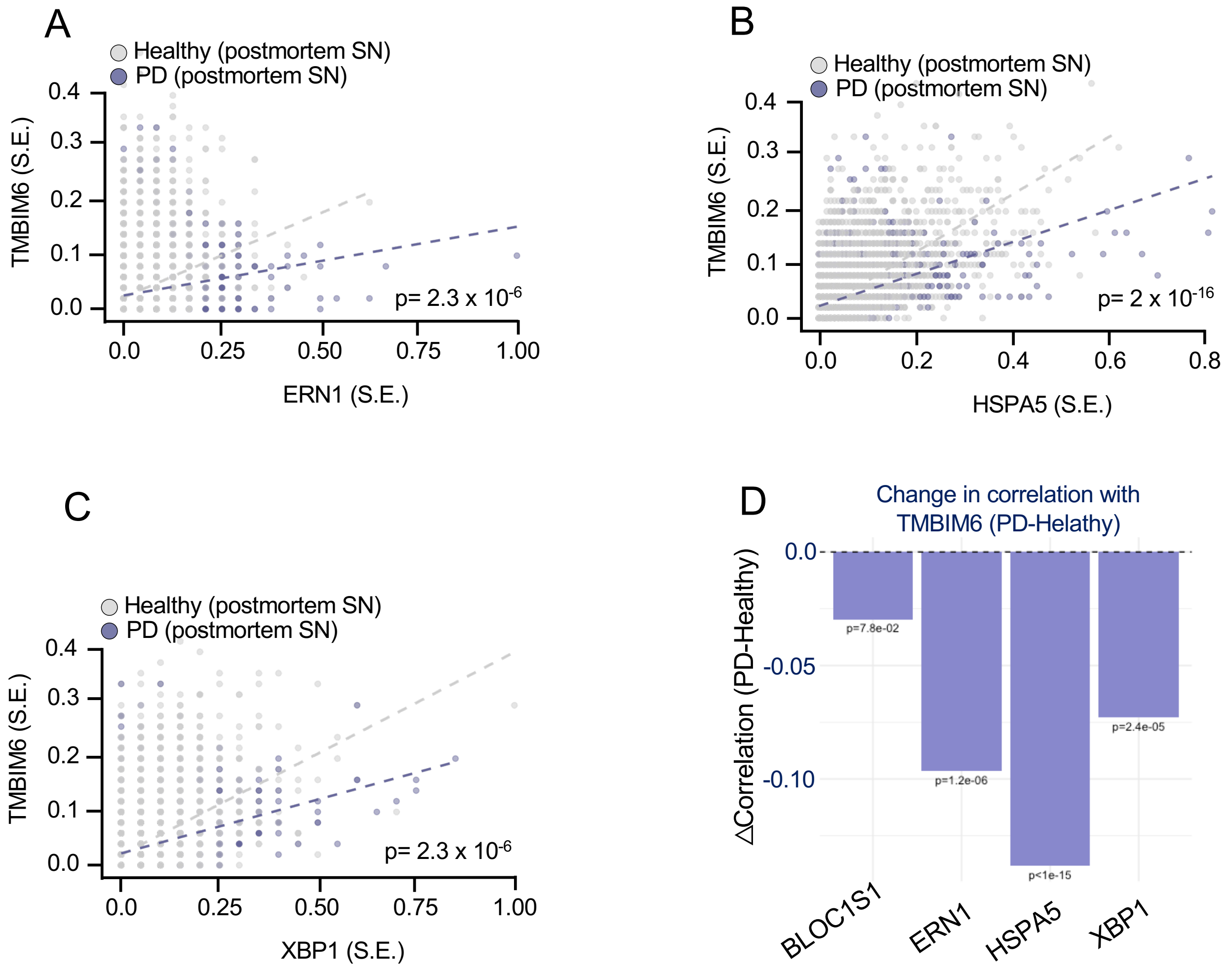

E

gene	cor_normal	cor_parkinson	delta_cor	z_score	p_value	adj_p_value	significant
BLOC1S1	0,4515818	0,4219351	-0,02965	1,761894	0,07808713	0,078087	No
ERN1	0,3116549	0,2151645	-0,09649	4,991032	6,00576E-07	1,2E-06	Yes
HSPA5	0,6258053	0,4875603	-0,13825	9,695586	2E-16	2E-16	Yes
XBP1	0,4629809	0,3900461	-0,07293	4,291277	1,77649E-05	2,37E-05	Yes

F

Gene	Comparison	logFC	mean_normal	mean_pd	pct_expr_ normal	pct_expr_pd	p_val_hurdle	adj_p_val_ hurdle	**p_val_cont**	p_val_disc
TMBIM6	Healthy - PD	0,00047364	1,472519765	1,57127072	58,9326702	62,8729282	0,00054384	0,0006798	0,99346841	0,00010561
ERN1	Healthy - PD	0,46645391	0,575809742	1,10460405	36,0112216	53,480663	1,681E-99	8,407E-99	1,3017E-37	3,7285E-65
XBP1	Healthy - PD	0,42475266	0,537745473	0,90570902	32,7913798	43,8674033	6,082E-49	1,5205E-48	1,6163E-23	2,1304E-28
HSPA5	Healthy - PD	0,33003404	3,171321092	3,70349908	62,1206325	68,1399632	2,4202E-09	4,0337E-09	0,08056653	1,4316E-09
BLOC1S1	Healthy - PD	0,11015128	0,338051517	0,33112339	22,6090283	20,626151	0,00292885	0,00292885	0,0118332	0,02095416

Gene	Comparison	logFC	mean_vulnerable	mean_resistant	dropout_ vulnerable	dropout_ resistant	p_val_hurdle	adj_p_val_ hurdle	**p_val_cont**	p_val_disc
TMBIM6	Vulnerable - Resistent	-1,1663465	1,41645093	2,28363807	41,6097905	36,4275668	5,6333E-49	9,3889E-49	4,5585E-46	1,1978E-05
ERN1	Vulnerable - Resistent	0,00309097	0,619675218	0,65494609	62,8971523	60,7126113	0,17735024	0,17735024	0,94713924	0,063066
XBP1	Vulnerable - Resistent	-0,2475515	0,540597788	0,69292077	67,0863733	63,3380216	3,901E-08	4,8762E-08	1,2486E-06	0,00111984
HSPA5	Vulnerable - Resistent	-3,5682885	2,161685102	5,14533521	42,7041657	29,9109236	3,2087E-91	1,6043E-90	1,2938E-66	8,2819E-28
BLOC1S1	Vulnerable - Resistent	-0,9887074	0,298305484	0,74308486	80,3130148	70,323488	2,3533E-50	5,8831E-50	5,9976E-31	2,1798E-22

Fig. S8. TMBIM6$^{D213A/HA}$ dissociates from IRE1 in response to aSyn PFFs

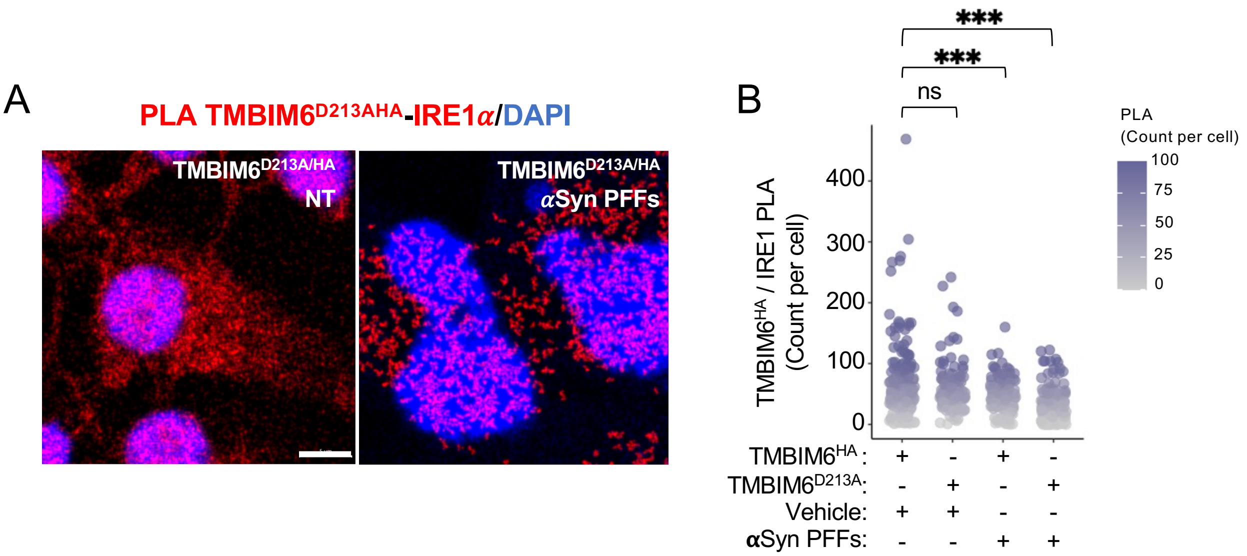

Fig. S9. Effect of the TMBIM6$^{C\text{-term}}$ on the TMBIM6–IRE1 complex and aSyn PFF-induced cell death

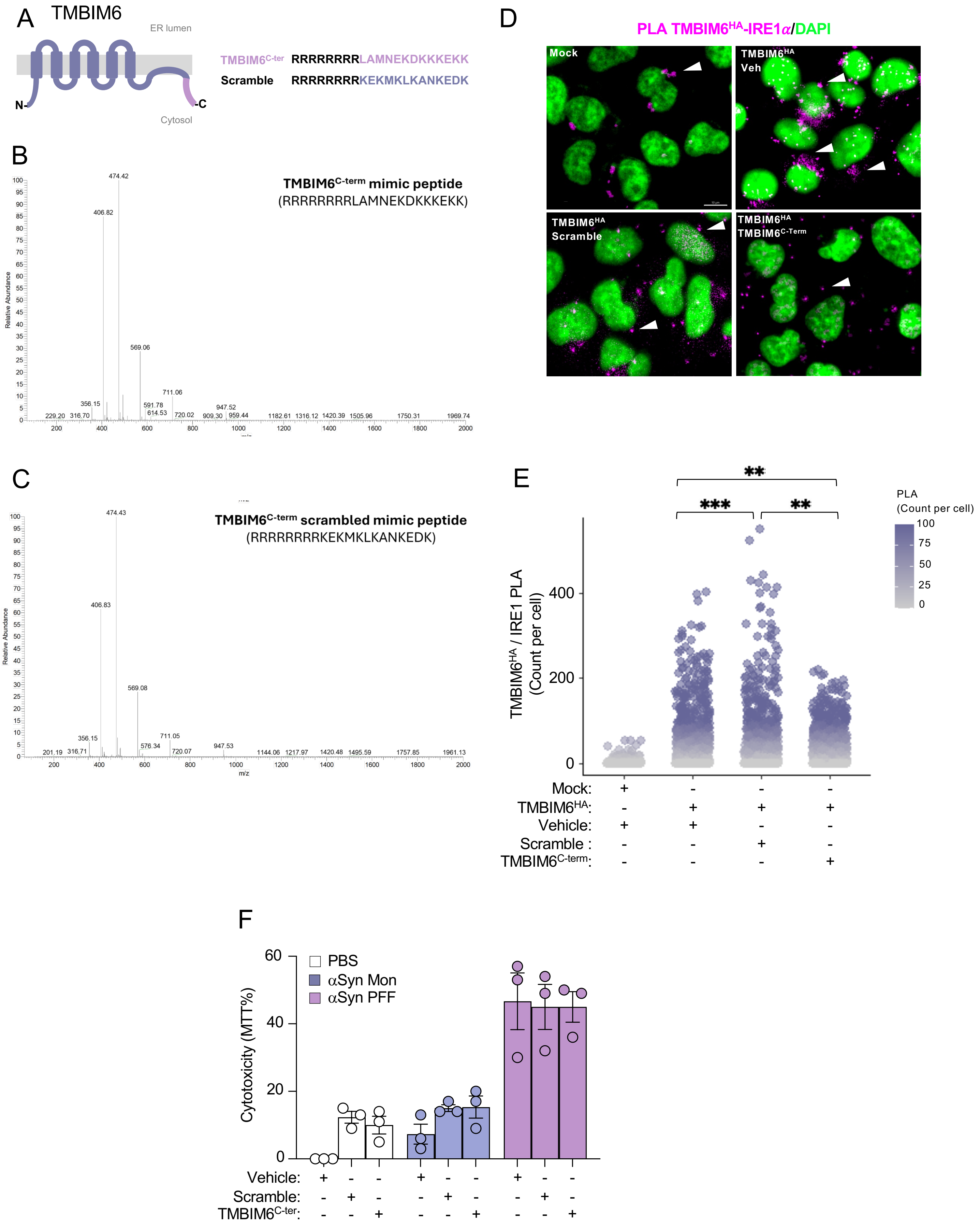

Fig. S10. Impact of the AAV-TMBIM6$^{HA/GFP}$ expression on *in vitro* models of PD

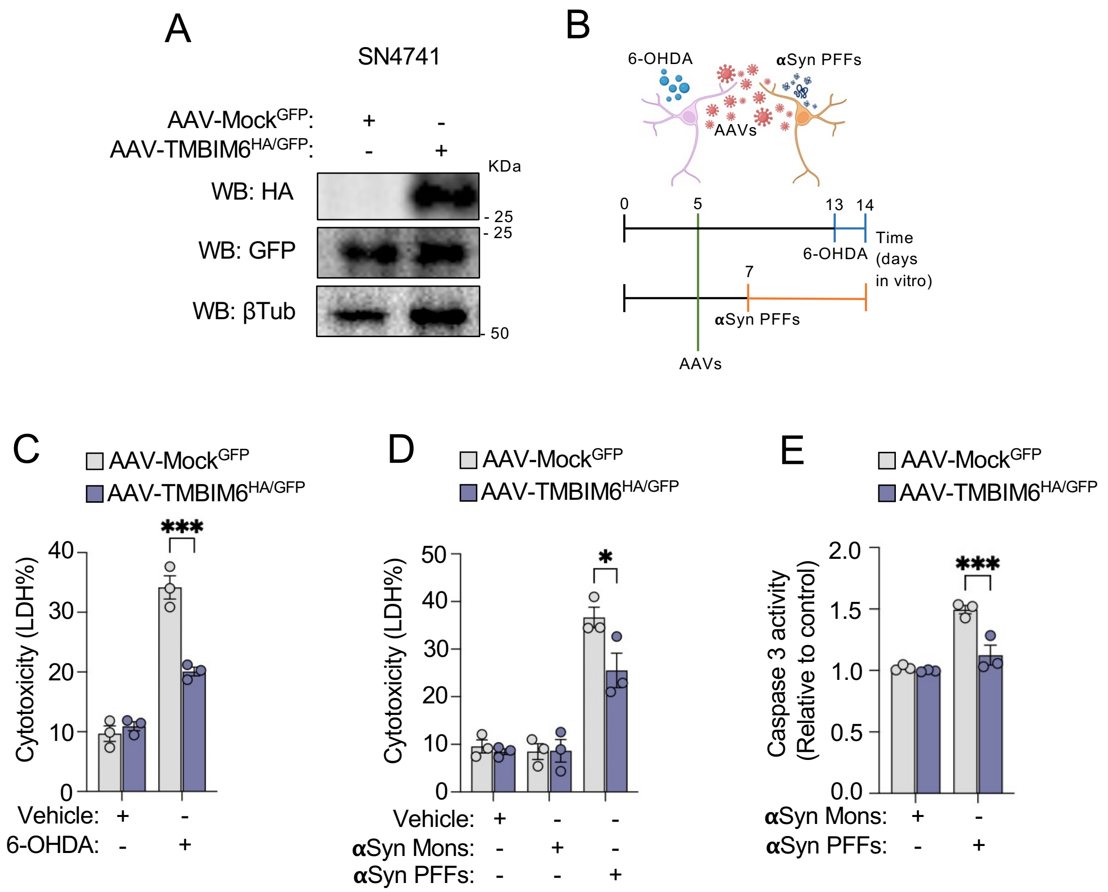

2B

si*Cntrl*: + -
si*Tmbim6*: - +
KDa

WB: TMBIM6

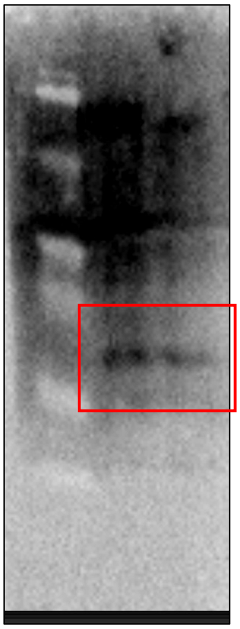

- 25

si*Cntrl*: + -
si*Tmbim6*: - +
KDa

WB: βTub

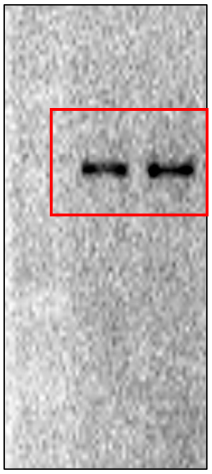

- 50

2J

si*Cntrl*: + - + - + -
si*Tmbim6*: - + - + - +
αSyn Monomers: - - + + - -
αSyn PFF: - - - - + +
Standard
KDa

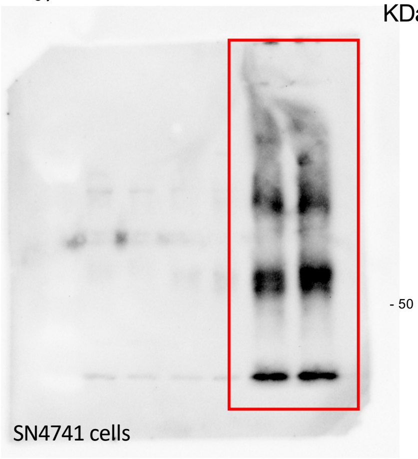

si*Cntrl*: + - + - + -
si*Tmbim6*: - + - + - +
αSyn Monomers: - - + + - -
αSyn PFF: - - - - + +
Standard
KDa

WB: βTub

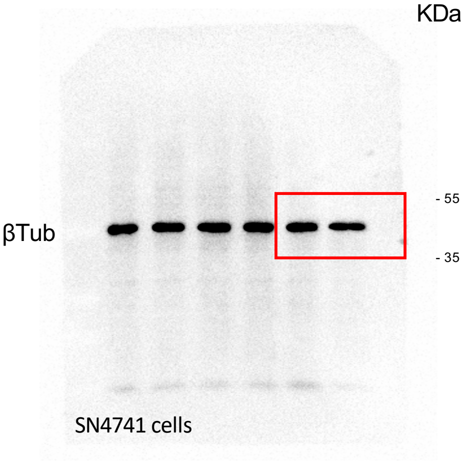

- 55
- 35

Ahumada-Montalva et al. 2025
Fig. S11. Westernblot membranes (Continuation)

4A

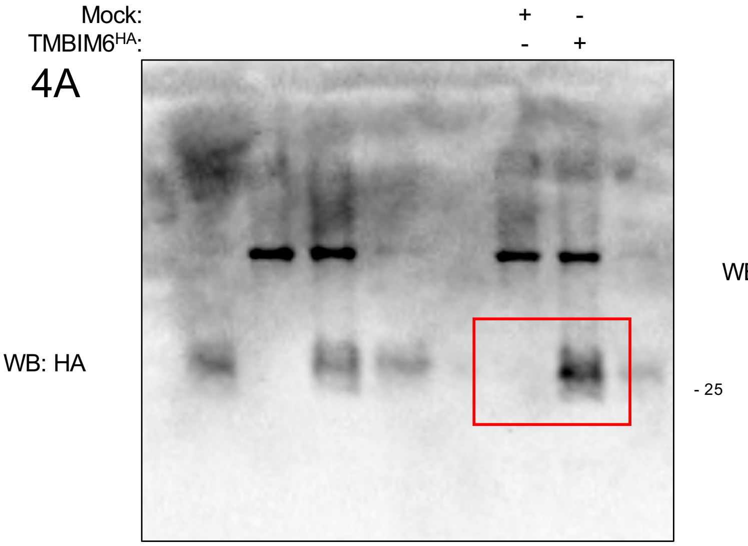

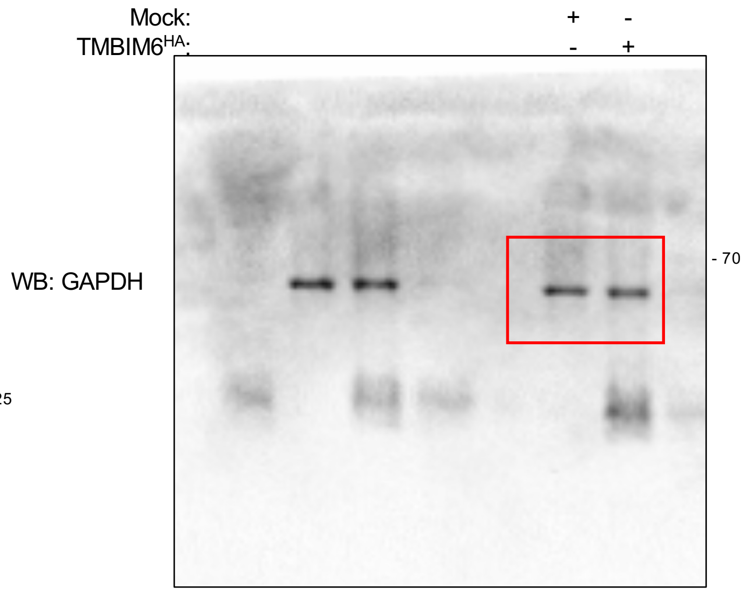

4I

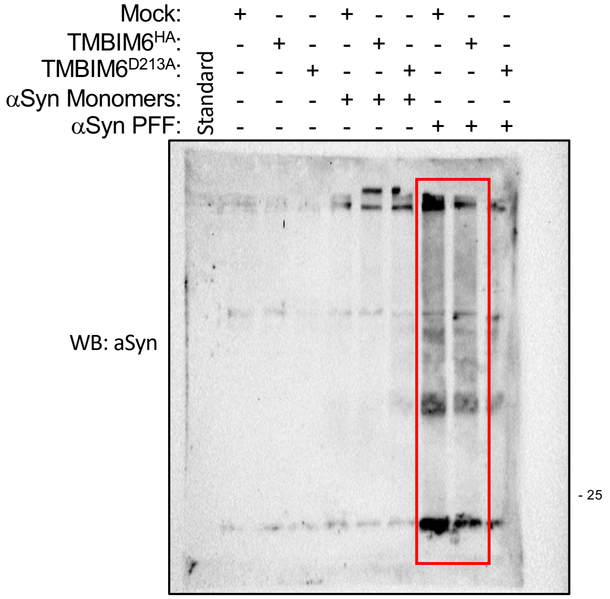

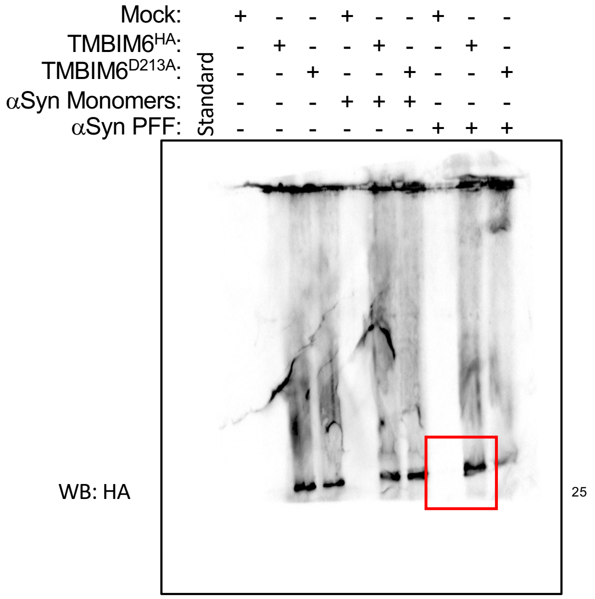

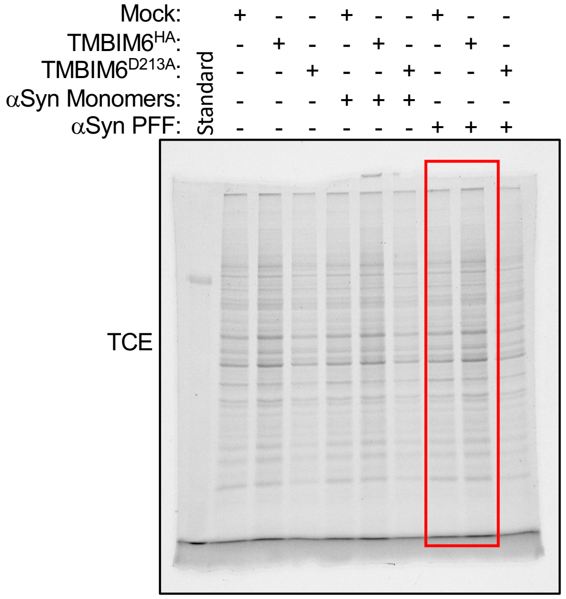

4K

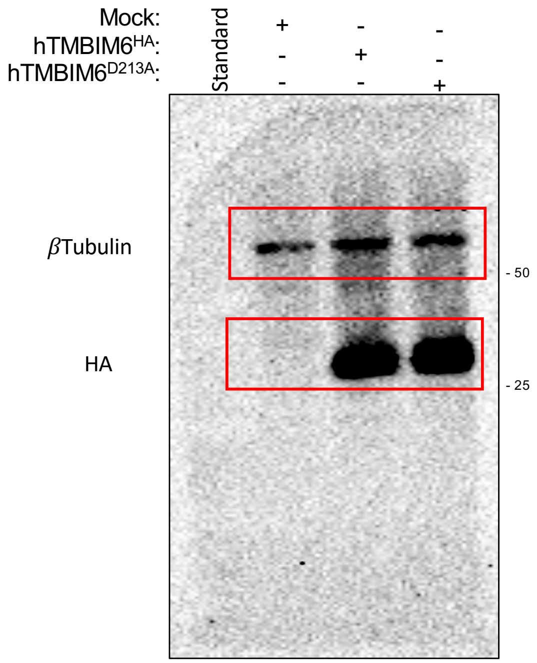

Sup. 6AB

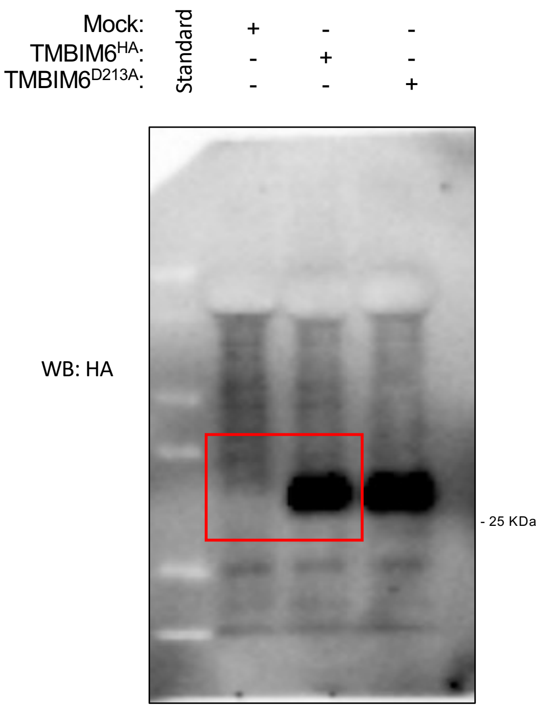

Primary cortical neurons

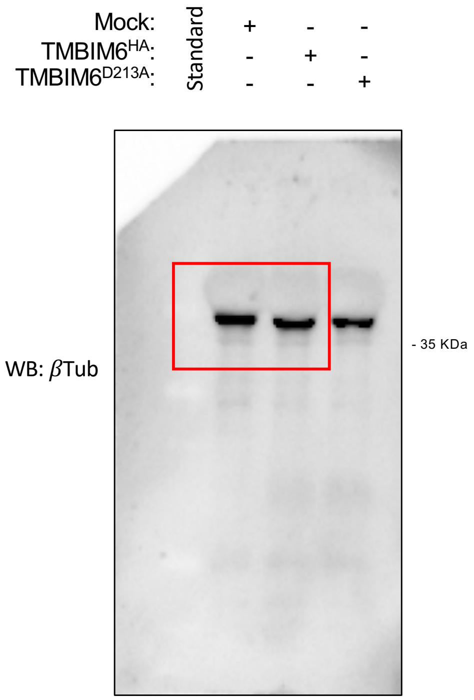

Primary cortical neurons

Sup. 10A

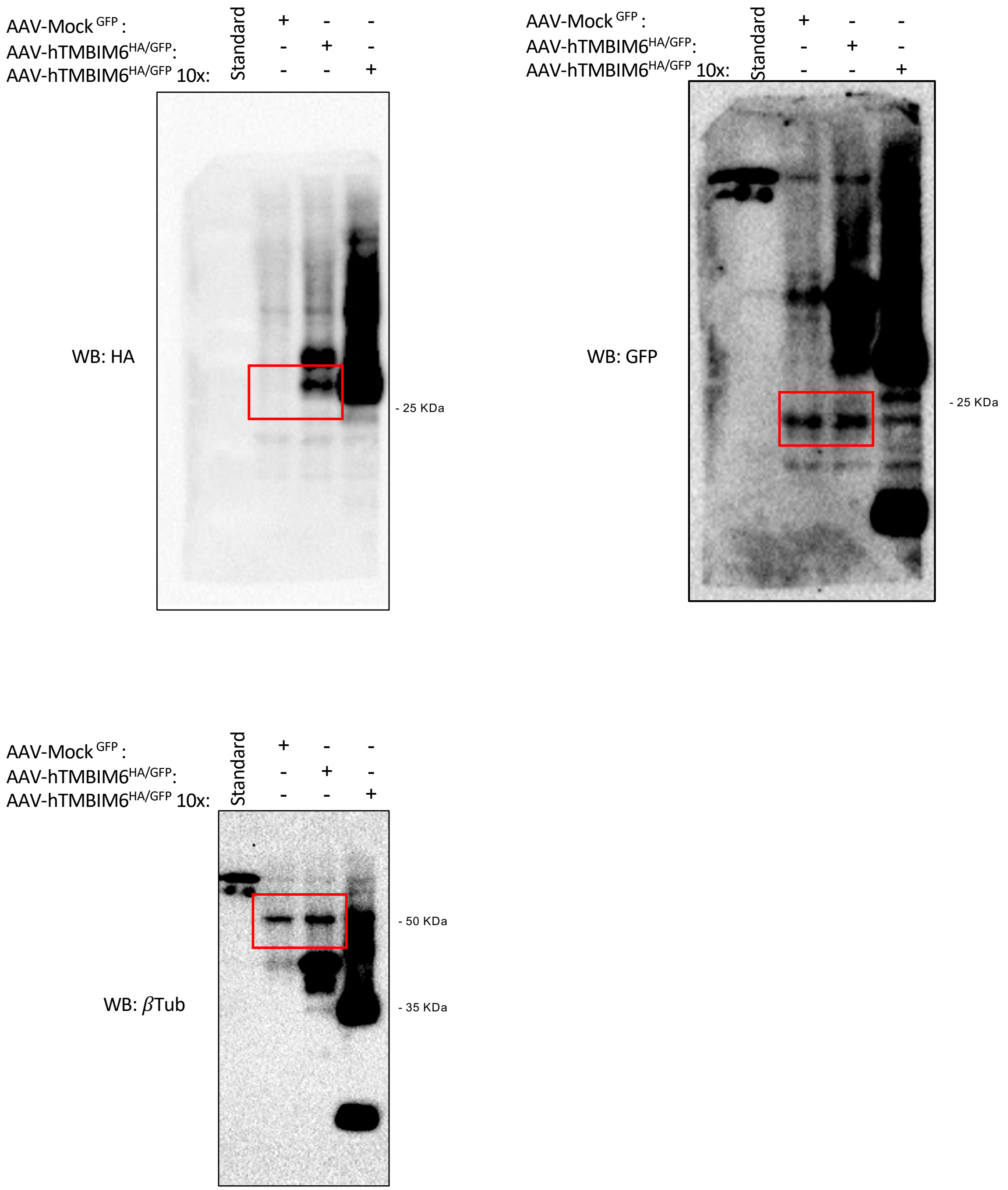
AAV-Mock GFP :
AAV-hTMBIM6HA/GFP:
AAV-hTMBIM6HA/GFP 10x:
Standard
+ - -
- + -
- - +
WB: HA
- 25 KDa
AAV-Mock GFP :
AAV-hTMBIM6HA/GFP:
AAV-hTMBIM6HA/GFP 10x:
Standard
+ - -
- + -
- - +
WB: GFP
- 25 KDa
AAV-Mock GFP :
AAV-hTMBIM6HA/GFP:
AAV-hTMBIM6HA/GFP 10x:
Standard
+ - -
- + -
- - +
WB: βTub
- 50 KDa
- 35 KDa
